# Coupled Cluster Semiclassical Estimates of Experimental Reaction Rates: The Interconversion of Glycine Conformer VIp to Ip

**DOI:** 10.1021/acs.jpclett.3c02560

**Published:** 2023-10-31

**Authors:** Giacomo Mandelli, Luca Corneo, Chiara Aieta

**Affiliations:** Dipartimento di Chimica, Università degli Studi di Milano, via C. Golgi 19, Milano 20133, Italy

## Abstract

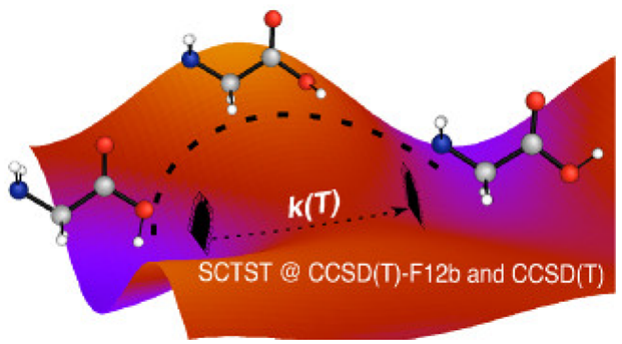

We apply the full-dimensional Semiclassical Transition State Theory (SCTST) to estimate the rate constant of glycine molecule interconversion between the VIp and Ip conformers. We have reached an electronic structure accuracy up to the explicitly correlated Coupled Cluster method (CCSD(T)-F12b/cc-pVDZ-F12) thanks to our parallel implementation. The reaction has been experimentally investigated in the literature and is known to proceed by quantum mechanical tunneling. The SCTST rates improve over other theoretical methods, and our results align with the experimental measurements, thus confirming the accuracy of the fully coupled anharmonic semiclassical tunneling treatment, providing that the level of electronic structure theory gives a reliable estimate of the reaction barrier height and shape. The comparison with experimental half-life times supports the validity of SCTST for glycine VIp–Ip conformer conversion in the cryogenic temperature range, where this theory is usually not considered applicable due to the onset of the deep tunneling regime.

To establish a synergy between experiments and theory and obtain mechanistic information and reliable interpretations of chemical reactions, two aspects must be considered for a suitable computational approach: First, the inclusion of nuclear quantum effects such as tunneling and Zero Point Energy (ZPE), since these are essential to calculate reaction rates at cryogenic and even at room temperature.^1−4^ Second, if it is required to compute accurate estimates of experimental rates, then it may be necessary to employ a high level of electronic structure theory.

This work focuses on glycine, a pivotal molecule in chemistry and biology. It is the smallest and simplest amino acid. Functioning as a fundamental constituent of proteins, it may clarify the origin of life as a molecule within the interstellar medium.^5,6^

Despite glycine’s simple structure, its conformational analysis is a complex task due to its ability to adopt multiple conformations.^7−11^ These conformations have been studied theoretically and experimentally during the past years.^12−14^ The high energy conformer VIp is peculiar among them because it is an elusive conformer due to its very short lifetime from the experimental point of view, even under low temperature and darkness conditions. Its experimental observation was possible thanks to the near-infrared laser light irradiation at the first OH or NH stretching overtone bands of more stable matrix-isolated glycine conformers.^15^ Thus, an investigation of VIp glycine conformer reactivity has been prompted. Isomer VIp converts to the global minimum Ip conformer on the glycine Potential Energy Surface (PES) with an isomerization path. This conversion is schematically represented in Figure 1. The reaction coordinate is close to a rigid rotation of the OH group around the principal CO axis of the molecule, and this large amplitude motion could be fairly anharmonic and coupled to other modes.

**Figure 1:**
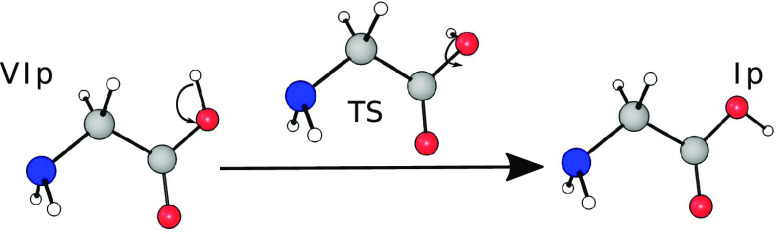
Interconversion reaction from the glycine conformer VIp to Ip. The main contribution to the reaction coordinate is given by the OH moiety rotation.

Bazsó et al.^15^ have observed this phenomenon experimentally with a detailed kinetic study in three different noble gas matrixes. They found the VIp conformer apparent instability is due to its fast decay toward the Ip one. Their study indicates the following half-life times at 12 K: 2.8 ± 1.0 s in xenon, 4.0 ± 1.0 s in krypton, and 4.4 ± 1.0 s in argon. The same reaction was also conducted with isotopic substitutions, thus replacing the two amine group hydrogen atoms and the hydroxyl group hydrogen atom with deuterium. In the trideuterated (TD) glycine case, the rates at 12 K are the following: 48.0 ± 1.0 h in krypton, 50.0 ± 1.0 h in argon, and 99.3 ± 2.0 h in xenon. Given the obtained rates at cryogenic temperatures and the deuteration studies, the interconversion reaction between the VIp and Ip isomers is thought to undergo hydrogen tunneling, which would explain the very fast disappearance of the VIp conformer.

In the present work, we propose to estimate the rate of interconversion of isomer VIp to isomer Ip of the isolated glycine molecule using a multidimensional, fully coupled, and nonseparable rate theory capable of capturing nuclear quantum effects, including tunneling. A technique that falls into this category is the Semiclassical Transition State Theory (SCTST) developed in the 1970s and later in the 1990s by W. H. Miller et al.^16,17^ In recent years, our group developed and applied^4,18,19^ SCTST to study tunneling phenomena in organic chemistry and confirm other experimental results which suggest the presence of room temperature heavy atom tunneling. Here, we show that the SCTST can provide experimentally accurate rates at cryogenic temperatures for reactions of medium to high dimensionality. Moreover, our parallel implementation distributed with the Multiwell program suite^4,18,20,21^ makes it practical to use high levels ab initio theories such as the CCSD(T) or CCSD(T)-F12b, which allow us to compare theoretical results with experimental reaction rates reducing any ab initio bias.

The SCTST is a convenient theory as it requires only simple quantities, such as the reaction barrier, the harmonic frequencies of the reactant and the transition state, and the anharmonic couplings using the Second-order Vibrational Perturbation Theory (VPT2). The SCTST working formula is the following:
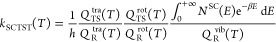
1where *Q*_TS(R)_^tra^(*T*) and *Q*_TS(R)_^rot^(*T*) are the translational and rotational free motion partition functions for the transition state (reactant). In our calculations, *Q*_R_^vib^(*T*) is the fully coupled and anharmonic VPT2 vibrational partition function for the reactant molecule, and it is recovered from the reactant vibrational Density Of States (DOS) ρ(*E*) as *Q*_R_^vib^(*T*) = ∫_0_^+*∞*^ρ(*E*) e^–*βE*^ d*E*. *N*^SC^(*E*) is the Cumulative Reaction Probability (CRP) in the semiclassical approximation. The lower bound of the integration in eq 1 corresponds to the reactant ZPE.^16,22,23^ To compute *N*^SC^(*E*), Miller and Hernandez^24^ exploited the inversion of the standard VPT2 energy expression^25^ in terms of the vibrational quantum numbers *n*_*k*_ extended to the case of a transition state on the PES^24^
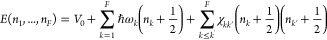
2to obtain a multidimensional generalization of the WKB barrier penetration integral. In eq 2, for a system with *F* vibrational degrees of freedom, *V*_0_ is the potential energy at the stationary point of the PES with the inclusion of a constant  term arising from the derivation of this expression in VPT2 context, ω_*k*_ is the harmonic frequency of the *k*-th normal mode, the terms χ_*kk′*_ are the anharmonic couplings whose expressions are given in the Supporting Information.

We start by computing the forward barrier heights at different levels of ab initio theory, as shown in Figure 2. In terms of electronic energy, all our forward energy barrier estimates lie within a 2 kJ/mol range. The Biczysko et al.^12^ barrier obtained with a composite CCSD(T)/(CBS+CV) scheme is also included within this range. However, their TS geometries are estimated at the B2LYP-D3BJ/aug-cc-pVTZ (aVTZ) level, while our optimizations and energies are calculated at the same ab initio level. Considering the aVDZ basis set, the DFT methods (B3LYP, B2PLYP-D3BJ, and B3LYPD3) give barrier estimates closer to each other than the post-Hartree–Fock methods (MP2, CCSD, and CCSD(T)). To improve the consistency between the barrier estimates, we repeat the calculation with the larger aVTZ basis set (green lines) and also with the CCSD(T)-F12b/VDZ-F12 model (purple line), obtaining a 0.56 kJ/mol agreement between the different ab initio methods.

**Figure 2:**
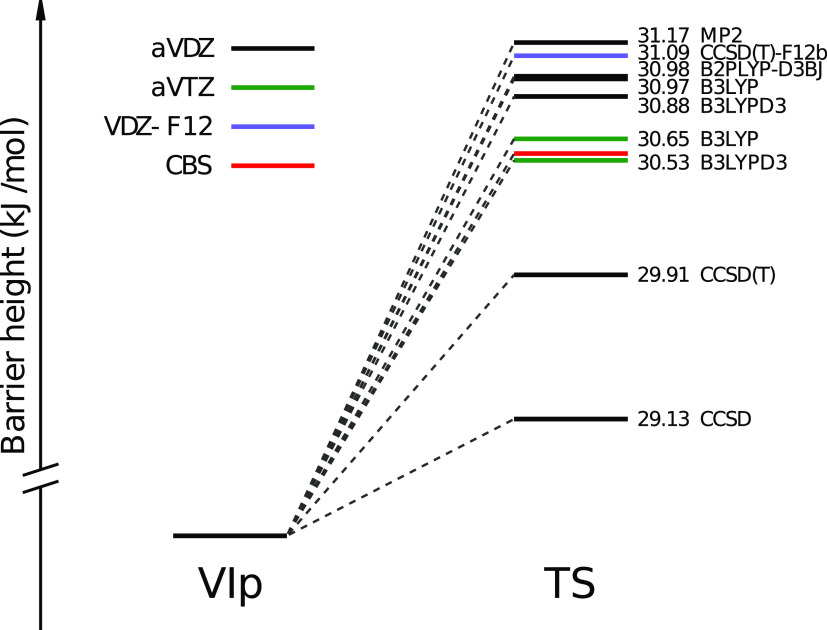
Glycine VIp to Ip interconversion forward electronic energy barrier heights. Geometry optimizations and energy calculations are computed at the same level of theory and with the same basis set. The red line refers to the Biczysko et al. estimate.^12^

After verifying the accuracy of the forward reaction electronic energy barriers compared to the benchmark results and ensuring convergence in the calculation of the anharmonic couplings, we proceeded to compute the SCTST reaction rates. Two reaction channels are involved in the VIp–Ip isomerization reaction, as the TS involved has two enantiomers and hence two possible conformations at the same energy. Therefore, it is necessary to divide the lifetime by a factor of 2, and the SCTST half-life time for the isomer VIp is calculated as follows:
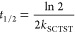
3where *k*_SCTST_ is computed using eq 1.

We report in Figure 3 the comparison between the classical and the SCTST results for the nondeuterated (ND) glycine reaction at two selected levels of theory: B3LYPD3/aVTZ and CCSD(T)-F12b/VDZ-F12. The classical and SCTST rates converge asymptotically in the high-temperature range. In Figure 3, the non-negligible difference between the two rate estimates highlights the importance of having a reaction rate theory that can be applied using high-level electronic structure theories (without a fitted potential energy surface). The conspicuous deviation from linearity in the considered temperature range can be attributed to both quantum effects and anharmonicities.

**Figure 3:**
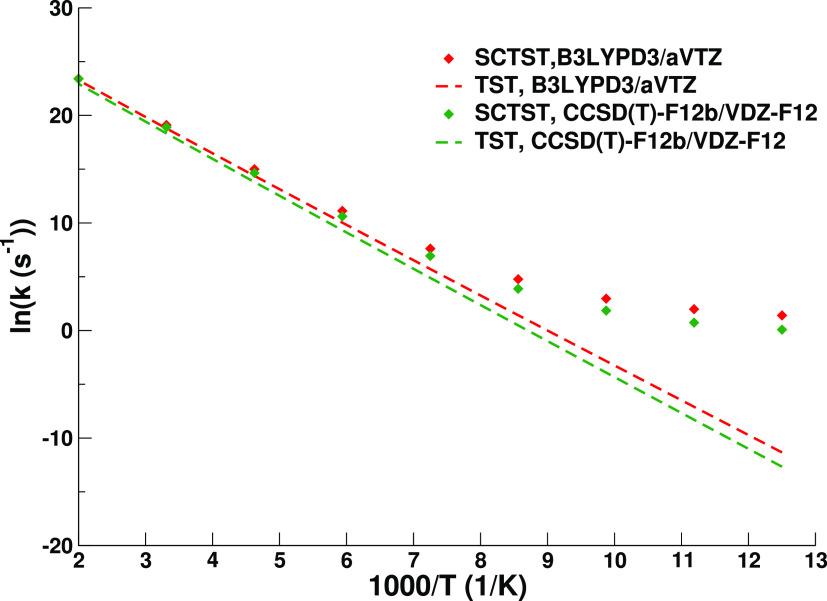
SCTST and TST reaction rates for the interconversion of ND glycine VIp to Ip. The B3LYPD3/aVTZ and CCSD(T)-F12b/VDZ-F12 rates are shown in temperatures ranging from 77 to 500 K.

In Figure 4 we plot the SCTST half-life time of the ND conformer VIp in a limited temperature range around the available experimental values at different levels of theory. Experimental results are available at 12 K. All our calculations with methods ranging from DFT to CCSD(T) give results less than 1 order of magnitude away from the experimental values in the three gas matrixes. The consistency among the SCTST rate constants can be rationalized by looking at the anharmonic χ_*kk′*_ coupling variations with the ab initio level of theory (see the Supporting Information). Indeed, despite the changes in the employed theoretical method and the forward barrier variations, we found a generally small percentage variation in the anharmonic coupling values. The CCSD/aVDZ, all of the B3LYP, and the CCSD(T)/aVDZ calculations provided estimates compatible with the experimental accuracy. The B2PLYP results underestimate the experimental results. Therefore, given the good performances and the general agreement among the other SCTST results, we can assume that the B2PLYP outlier may be caused by a problematic PES around the minimum or transition state geometry. The post-HF methods MP2/aVDZ and CCSD(T)-F12b/VDZ-F12 delivered results within a factor ∼2 over the experimental half-lives. As we will detail later, we think that the more reliable result is the CCSD(T)-F12b/VDZ-F12 one, and the overestimation of the experimental half-life time is due to the gas phase calculation that does not account for the cryogenic matrix environment. In Figure 4 we also report other authors’ results from theoretical approaches considering a one-dimensional reaction coordinate model (cyan symbols). Bazsó et al.^15^ used the Eckart model for tunneling through an asymmetric one-dimensional (1D) barrier.^20^ They carried out the calculations at the MP2/6-311++G** level of theory and obtained a half-life of 658 s at 12 K (2 orders of magnitude off the experiment). More recently, Bowman et al.^26^ fitted a DFT B3LYP/aug-cc-pVDZ (aVDZ) PES and computed the rate of isomerization by locating the tunneling path and using a 1D approach to compute the WKB penetration integral.^27^ Under these approximations, their estimate of the isomerization rate is 0.43 s (underestimating by 1 order of magnitude the experimental results). Then, they applied a morphing technique to adjust their 1D barrier height to the energy difference between the conformers and the TS optimized at the CCSD(T)/aVDZ level of theory (even if the authors point out that the CCSD(T) saddle point geometry did not fully converge). The adjusted higher barrier lengthens the VIp conformer half-life to 7 s. These 1D approaches are based on describing the reaction as a rigid rotation of the −OH group and approximating the involvement of other parts of the molecule by relaxing the geometry along the torsional PES cut. Instead, our SCTST fully coupled and anharmonic calculations are able to give accurate estimates of the half-life time using a high level of electronic structure theory without the need for a fitted potential energy surface and without resorting to one-dimensional models of the reaction coordinate.

**Figure 4:**
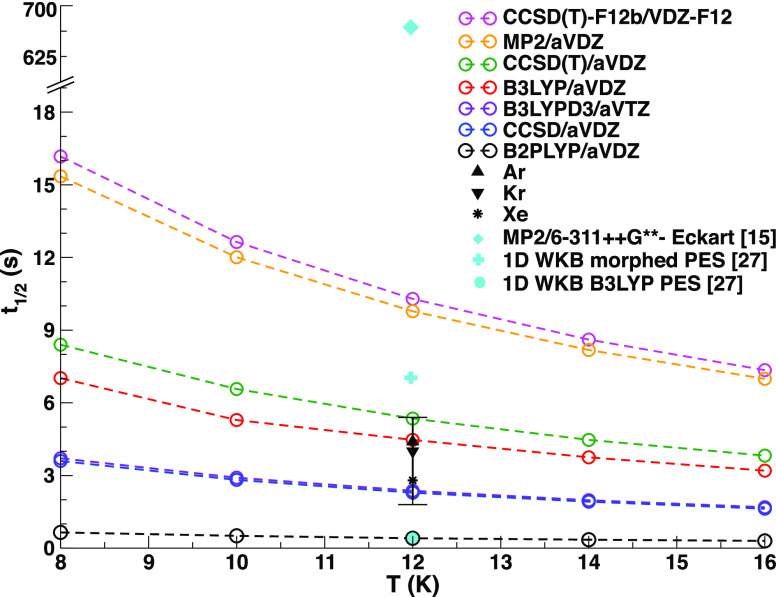
Dotted lines: Conformer VIp SCTST half-life times at temperatures ranging from 8 to 16 K at a different level of ab initio theory and BS. Vertical error bar: Uncertainty bar for the experimental results at 12 K in argon, krypton, and xenon matrixes. The cyan symbols indicate literature results from 1D reaction coordinate theoretical approaches.

To confirm the ability of our SCTST calculations to describe the tunneling in the VIp–Ip glycine conversion, we proceed with the trideuteration of the molecule, involving the OH and the two NH_2_ hydrogen atoms, as was done in the experiments. The trideuterated (TD) molecule shows a significant speed reduction in the interconversion reaction compared to the nondeuterated one. Other than being caused by classical effects of the increasing mass, such a phenomenon has been proven to be related to the quantum mechanical tunneling of the hydrogen atom. Specifically, deuteration causes a slight increase in the barrier height when considering anharmonic ZPE-corrected barriers (see the Supporting Information). However, most importantly, the tunneling contribution to the rate from the lowest level, which is the most important at cryogenic temperatures even in the TD case, will decrease if compared to that in the ND case. We can picture this with a 1D separable assumption of the reaction coordinate; if the ZPE is lower in the TD reactant molecule, then a thicker barrier must be penetrated. As a remark, we report in Figure 5, the percentage difference between the rates for the ND and the TD glycine molecules,
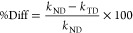
4both in the classical (TST) and the semiclassical (SCTST) case at the B3LYPD3/aVTZ level of theory. Indeed, the TST %Diff is only affected by classical contributions from the deuterium substitutions while the SCTST %Diff is also affected by changes in the quantum and anharmonic contributions related to the mass increase. As the temperature decreases, %Diff goes asymptotically to a constant value close to 0.5 in the TST case. The ZPE difference between the TD and the ND molecules causes this effect. In the SCTST case, instead, after an initial regime in which the %Diff almost linearly increases while the temperature decreases, a plateau is reached at a value close to 100%. Between 500 and 125 K, there is competition between over-the-barrier and tunneling processes, while below ∼125 K, tunneling starts to dominate as demonstrated by the temperature independence of the ND and TD rate constant difference. This is compatible with the harmonic estimate of the crossover temperature  which is an indication of the temperature at which tunneling and over-the-barrier reaction mechanisms are equally important.^28^ For the B3LYPD3/aVTZ model of the present reaction, *T*_c_ = 128 K.

**Figure 5:**
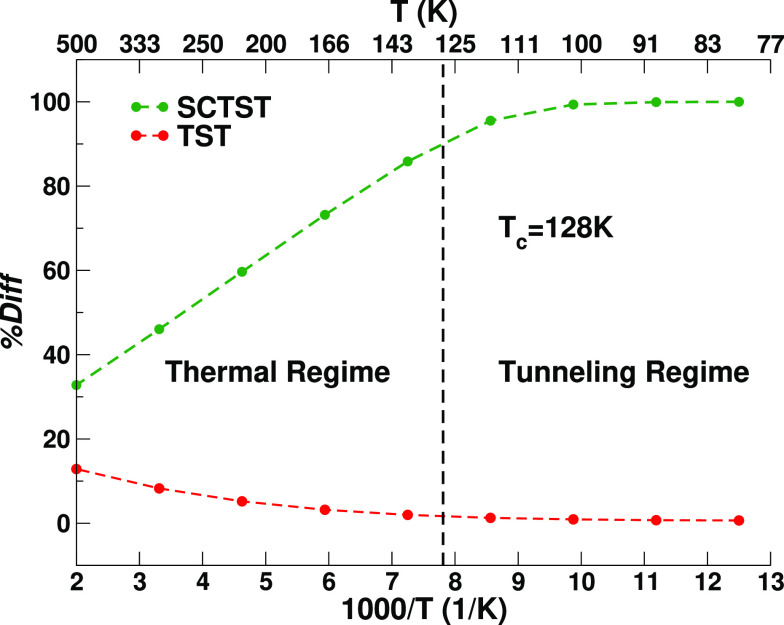
Percent difference between the trideuterated (TD) and nondeuterated (ND) rates for the classical TST and the quantum SCTST calculations in a temperature ranging from 77 to 500 K. Calculations are done at the B3LYPD3/aVTZ level of theory. The vertical dashed line indicates the crossover temperature *T*_c_ that marks the transition from the thermal to the tunneling regime.

At cryogenic temperatures, deuteration does not switch off the tunneling phenomena. The SCTST and the experimental results are shown in Figure 6. The B3LYPD3/aVTZ results in the gas phase lead to a 12 K half-life time estimate of the same order of magnitude as the experimental rates measured in different gas matrixes. The SCTST rates at the CCSD(T)/aVDZ level of theory for the TD molecule are instead slower (3.39 × 10^–7^ s^–1^) than the experimental values at 12 K (experimental range: 2.00 × 10^–6^ to 9.72 × 10^–7^ s^–1^). For the TD molecule with CCSD(T)-F12b/VDZ-F12, we obtained some anomalously large and negative terms for the couplings involving the lowest frequency normal mode, which hampers the SCTST calculation (see the couplings reported in the Supporting Information). With a larger step size for the finite difference derivative calculations, the anomalously large couplings are slightly reduced (but still quite large), giving a fully coupled half-life of 64 h at 12 K. Then, we decoupled the lowest frequency mode and considered it as a separate vibration, and the half-life time is close to that of the CCSD(T)/aVDZ one. All the explored approaches provide a half-life estimate within a factor of 3 of the experimental values, supporting that SCTST correctly models the TD molecule reaction. As in the ND molecule case, we regard the CC half-life calculations as the most reliable, confirming the hypothesis that gas phase estimates miss the effect of the matrix environment.

**Figure 6:**
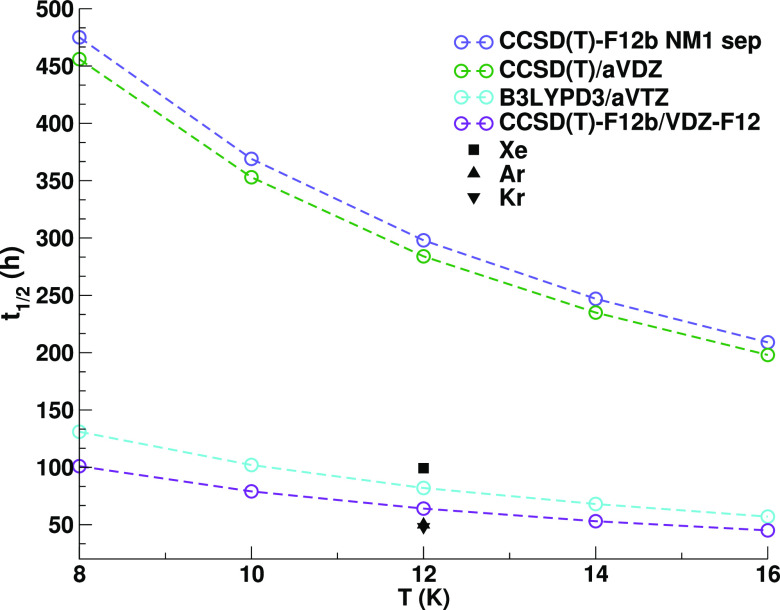
SCTST half-life times for the interconversion of TD glycine VIp to Ip. The B3LYPD3/aVTZ, CCSD(T)/aVDZ, and CCSD(T)-F12b/VDZ-F12 half-life times are shown in a temperature range from 8 to 16 K. The experimental results at 12 K in different gas matrixes are reported with black-filled symbols without error bars as they would not be visible on this time scale. The CCSD(T)-F12 values with the first normal mode separated in the calculation of the anharmonic constants are reported with the following label: CCSD(T)-F12 NM1 sep.

The computed SCTST half-life times show a dependence on the anharmonically corrected forward reaction barrier and the −i*ω*_*F*_ frequency of the reactive mode. Indeed, as shown in the Supporting Information, the general trend confirms that not only does the energy barrier play a role in estimating the rate constant but at cryogenic temperatures, the barrier width is also crucial. The latter can be estimated harmonically with the curvature at the top of the barrier using the imaginary frequency of the reactive mode. For example, considering the TD molecule in the B3LYPD3/aVTZ case, we have an imaginary frequency of 414i cm^–1^ and an anharmonic barrier of 27.28 kJ/mol while for the CCSD(T)/aVDZ we have a frequency of 397i cm^–1^ and a barrier of 26.90 kJ/mol. From these values and especially from the differences in the barrier width at the top, we expect the kinetic rate constant of the CC to be slower than the DFT one, despite the barrier being slightly higher for the DFT model. The CC rate constant is indeed 3.39 × 10^–7^ s^–1^ while the B3LYPD3 is 1.17 × 10^–6^ s^–1^. The compensation effect between the barrier height and its width can give an indication of the relative magnitude of the rate constant for two ab initio methods. This is just an estimate as the anharmonic couplings, as shown, play a fundamental role in assessing the final SCTST rate constants value.

As anticipated in the results presented above, the specific gas matrix affects the experimental rate constants. This effect is observed in other reactions, such as the formic acid conformer interconversion.^29^ This phenomenon may be caused by phononic interactions depending on the matrix used,^30^ and differences can also arise depending on the site occupied by the reacting molecule in the matrix.^7,31^ In the glycine case, this difference is emphasized in the TD experiment, where the rate constant in the xenon gas matrix is significantly slower than the other two matrixes. Our simulations are all carried out under vacuum conditions. Therefore, we expect the system behavior to be influenced by the absence of the environment. This can be the source of the difference between the experimental and CCSD(T)-F12b results. This aligns with the observation that the F12 rate constant value in vacuum agrees with the trend observed for the rate in different matrixes: the lighter the matrix, the slower the reaction (except for Xe matrix TD experimental result); see Figure 4. It will be interesting to investigate with a detailed study the effects of the medium on the SCTST reaction rates thanks to the ability of this approach to include the reaction coordinate coupling with the other modes. This topic has been deferred to a future study.

It is well-known that the SCTST rate theory can fail for reactions that proceed in the deep tunneling regime.^32^ The deep tunneling regime occurs when the temperature is very low, so that the reactant system level occupation is limited to states well below the top of the barrier. In general, the deeper the levels, the more anharmonic the shape of the barrier. In the present glycine VIp–Ip conformer conversion reaction study, the temperature is very low, but in this case, this is insufficient to claim that the SCTST becomes invalid. This has to be checked case by case. The diagonal coupling term for the reactive mode χ_*FF*_ is negative for the present reaction at all the considered electronic structure levels. As discussed by Barker and Wagner,^32,33^ the deep tunneling failure of SCTST occurs when the multidimensional barrier penetration integral  becomes complex, that is, when Δ*E* > *D* = −ℏ^2^Ω_*F*_^2^/4χ_*FF*_. In these expressions −Δ*E* is the vibrational energy available along the reaction coordinate and ℏΩ_*F*_ is the effective reaction coordinate frequency (see, for instance, ref (32) for explicit definitions Δ*E* and ℏΩ_*F*_). We numerically monitored that this never happened in our calculations for both the ND and the TD cases. We additionally implemented the Wagner deep tunneling SCTST correction and employed it in the B3LYPD3/aVTZ case as an example. As expected, it does not significantly impact the calculated half-life time values (see the Supporting Information). The TD rates are slightly more sensitive to the inclusion of the Wagner correction because the tunneling is deeper in the TD case due to the lower ZPE.

In conclusion, in this work, we have pushed the boundaries of the reaction rate calculations state-of-the-art in terms of both nuclear quantum effects treatment and the underlying ab initio level of theory. We have shown that with SCTST it is possible to predict experimental reaction rates of glycine conformer interconversion, including full-dimensional quantum effects and full anharmonic coupling of all reactive and bound modes. Thanks to our SCTST implementation, we reached the CCSD(T)-F12b/VDZ-F12 level of theory without a fitted PES, and all our estimates are within a factor of 2 (3 for the TD case) compared to experimental values. We shed light on the capabilities of SCTST to venture into the cryogenic temperature regime. Specifically, we numerically assessed the validity of SCTST for glycine conformer interconversion at cryogenic temperature. We also think it is valuable to confirm or reassess available glycine interconversion rate estimates with the SCTST approach to analyze which key physical effects contribute to the accuracy of the predictions. Future work will focus on explicitly considering the matrix environment and investigating the abundant literature reaction rate data measured in rare gas matrixes at cryogenic temperatures, such as the formic acid rotamerization.^29^

## Methods

To compute the anharmonic couplings, we used our FDACC script^20^ interfaced with the Gaussian16 software^34^ for the optimization, the Hessian and the Single Point Energy (SPE) calculations. For the Coupled-Cluster (CC) calculations using the CCSD(T)-F12b method with the VDZ-F12 BS, we interfaced the FDACC program with the MOLPRO software.^35^ With this explicitly F12 correlated method,^36,37^ the accuracy of the results with the VDZ-F12 BS reaches at least the conventional aVQZ quality. Especially for this system with a flat and quartic minimum for the VIp conformer, it is necessary to be cautious when optimizing the structures. Indeed, when the second derivative matrix is computed, it is essential to verify that the eigenvalues associated with rotation and translation motions are small enough. Furthermore, extra care must be taken in evaluating the stability of ab initio calculations in terms of the TS anharmonic analysis. For our calculations, we checked the convergence of the rates with the finite difference step size that we employed for the potential derivatives necessary for the anharmonic couplings. We found a 0.5–0.4 step size (for its definition, see eq 26 in ref (4)) delivers reliable results for all ab initio methods considered here. The FDACC script implements the second-order vibrational perturbation theory plus resonances (VPT2+K) expression to account for accidental frequency degeneracy.^4,38^ For the density of states calculation, the barrier penetration integrals necessary for the SCTST are obtained with the paradensum and parsctst programs of the Multiwell program suite.^18,19^ In our calculation, we also included the barrier frequency correction factor for the F-th reactive mode  as suggested in ref (39). The details about the parameters and the input used can be found in the Supporting Information.

## Supporting Information Available

Optimized geometries for all levels of theory considered, the explicit formula for the anharmonic couplings, numerical values of harmonic and anharmonic barriers, imaginary frequencies and TST and SCTST rate values and half-life times at all levels of electronic structure theory considered, reaction rates with Wagner deep tunneling correction, and the values of the harmonic and anharmonic coupling in the paradensum and the parsctst programs input form (PDF)
