# Supplementary material for: Coupled Cluster Semiclassical Estimates of Experimental Reaction Rates: The Interconversion of Glycine Conformer VIp to Ip
Source: J Phys Chem Lett. 2023 Oct 31;14(44):9996–10002. doi: 10.1021/acs.jpclett.3c02560 (PMC10641884; doi:10.1021/acs.jpclett.3c02560)
Supplement: Supplementary file 1 — jz3c02560_si_001.pdf [file jz3c02560_si_001.pdf]

SUPPORTING INFORMATION for Coupled Cluster Semiclassical Estimates of Experimental Reaction Rates: The Interconversion of Glycine Conformer VIp to Ip

Giacomo Mandelli, Luca Corneo, and Chiara Aieta*

Università degli Studi di Milano, Dipartimento di Chimica, Via Golgi, 19 – 20133 Milano (Italy)

E-mail: chiara.aieta@unimi.it

Contents

Optimized Geometries **S3**

Anharmonic couplings **S6**

Calculated Rates for Glycine **S7**

Calculated Rates for Trideuterated Glycine **S11**

Wagner DT corrected rates **S12**

Frequencies and Anharmonicities for Glycine **S13**

Frequencies and Anharmonicities for tri-deuterated Glycine **S35**

Optimized Geometries

The optimization has been carried out with the Gaussian16 software for all geometries, with the exception of CCSD(T)-F12b where the MOLPRO software has been used instead. The convergence criteria have been set with the keyword OPT(VeryTight) for the software Gaussian, and for consistency, the same criteria have been replicated on MOLPRO using the string

```
{optg, GAUSSIAN, GRMS=1E-6, SRMS=6E-6, STEP=6E-6, GRADIENT=2E-6}
```

in the Molpro inputs

B3LYP/aVDZ

```
VIp, ENERGY=-284.474335611
N   -2.0141160125    0.0147323557    0.0000000000
H   -2.0583671187   -0.5990466367    0.8107890000
H   -2.0583671187   -0.5990466367   -0.8107890000
C   -0.7658088851    0.7507241397    0.0000000000
H   -0.7350877707    1.4117661343    0.8815470000
H   -0.7350877707    1.4117661343   -0.8815470000
C    0.5105259680   -0.0983350812    0.0000000000
O    0.5279657596   -1.3024010842    0.0000000000
O    1.6833840888    0.5997677159    0.0000000000
H    1.5156222537    1.5532917449    0.0000000000
```

```
TS, ENERGY=-284.462537905
N   -2.0254069509   -0.0343957092    0.0070593753
H   -2.0950979717    0.5802852553   -0.8010236499
H   -2.0840948934    0.5720023305    0.8222943500
C   -0.7605859726   -0.7435287474   -0.0081716529
H   -0.6996280348   -1.3859977907   -0.8998506259
H   -0.7093789516   -1.4343007090    0.8490463768
C    0.5011770535    0.1096182160    0.0089562464
O    0.5240110887    1.3103662150    0.0018121895
O    1.6719320280   -0.6420998218   -0.0584727753
H    1.9674370625   -0.9121007896    0.8213522230
```

B3LYP/aVTZ

```
VIp, ENERGY=-284.547697797
N    2.0097211066    0.0144220214    0.0000000000
H    2.0640191417   -0.5915796294    0.8094490000
H    2.0640191417   -0.5915796294   -0.8094490000
C    0.7635573957    0.7456941440    0.0000000000
H    0.7298774455    1.4033065421    0.8740470000
H    0.7298774455    1.4033065421   -0.8740470000
C   -0.5111132273   -0.1017234693    0.0000000000
O   -0.5311518974   -1.2984739097    0.0000000000
O   -1.6750624810    0.6026178313    0.0000000000
H   -1.5031444080    1.5522644899    0.0000000000
```

```
TS, ENERGY=-284.536024099
N   -2.0210570354   -0.0339781301   -0.0071016705
H   -2.0995526111    0.5738639315    0.7987677529
H   -2.0896170528    0.5635472217   -0.8215864335
C   -0.7587917190   -0.7395247366    0.0084305283
H   -0.6965660267   -1.3772875691    0.8935554466
H   -0.7061864782   -1.4273108600   -0.8408044684
C    0.5013878127    0.1120464344   -0.0091668086
O    0.5254901979    1.3055193034   -0.0018813756
O    1.6656240289   -0.6420231698    0.0581187796
H    1.9634847613   -0.9196063555   -0.8150223510
```

B3LYPD3/aVDZ

```
VIp, ENERGY=-284.479598297
N    2.0113737196    0.0160675037    0.0000000000
H    2.0491287433   -0.5997063689    0.8098660000
H    2.0491287433   -0.5997063689   -0.8098660000
C    0.7642419489    0.7550326025    0.0000000000
H    0.7314465212    1.4155414016    0.8819010000
H    0.7314465212    1.4155414016   -0.8819010000
C   -0.5100535978   -0.0977862916    0.0000000000
O   -0.5198081326   -1.3019501205    0.0000000000
O   -1.6861376895    0.5943888467    0.0000000000
H   -1.5244937018    1.5488846566    0.0000000000
```

```
TS, ENERGY=-284.467837022
N    2.0227238233    0.0356704298    0.0077681867
H    2.0872936853   -0.5814113695   -0.7991566089
H    2.0747201171   -0.5723504798    0.8224901484
C    0.7584072291    0.7466557668   -0.0089370452
H    0.6952249532    1.3885004308   -0.9009290143
H    0.7030078986    1.4368607695    0.8485005861
C   -0.5006046847   -0.1098518888    0.0078229509
O   -0.5180672152   -1.3106396610    0.0017377544
O   -1.6727004263    0.6392735220   -0.0577892446
H   -1.9652153233    0.9055455786    0.8240056425
```

B3LYPD3/aVTZ

```
VIp, ENERGY=-284.552949035
N    2.0070038898    0.0157414847    0.0000000000
H    2.0548223191   -0.5923499686    0.8085430000
H    2.0548223191   -0.5923499686   -0.8085430000
C    0.7620321788    0.7500670945    0.0000000000
H    0.7264792202    1.4071436355    0.8743890000
H    0.7264792202    1.4071436355   -0.8743890000
C   -0.5105920691   -0.1011158020    0.0000000000
O   -0.5229695586   -1.2979787773    0.0000000000
O   -1.6779297964    0.5971230819    0.0000000000
H   -1.5125985403    1.5478421143    0.0000000000
```

```
TS, ENERGY=-284.541319597
N   -2.0184382283    0.0352146110   -0.0078105793
H   -2.0919351547   -0.5750476864    0.7969374902
H   -2.0803681941   -0.5640517775   -0.8217783132
C   -0.7566513723    0.7425946156    0.0091975111
H   -0.6923274455    1.3797598012    0.8946120296
H   -0.6999798025    1.4298500215   -0.8402200879
C    0.5007880113   -0.1122680032   -0.0080602192
O    0.5197071597   -1.3057787933   -0.0018027215
O    1.6663725015    0.6392600258    0.0574464734
H    1.9613458183    0.9131602387   -0.8176779491
```

B2PLYP-D3BJ/aVDZ

```
VIp, ENERGY=-283.940416082
N   -2.0112058653   -0.0170459513   -0.0000002730
H   -2.0443262737    0.5996920488    0.8091284653
H   -2.0443273855    0.5996873332   -0.8091340795
C   -0.7625003160   -0.7545829466    0.0000007064
H   -0.7301855375   -1.4133464908    0.8821267088
H   -0.7301851252   -1.4133492073   -0.8821217371
C    0.5082341791    0.0982706901   -0.0000001617
O    0.5214824783    1.3061048372    0.0000002873
O    1.6845151923   -0.5985580835   -0.0000003674
H    1.5101385706   -1.5505346789   -0.0000007789
```

```
TS, ENERGY=-283.929391011
N   -2.0223612040    0.0369295617   -0.0069542360
H   -2.0817109620   -0.5798856185    0.8003720737
H   -2.0699605393   -0.5737201870   -0.8197800433
C   -0.7572969247    0.7477321502    0.0079180769
H   -0.6949502450    1.3886170940    0.8994813287
H   -0.7042658116    1.4346002511   -0.8511194304
C    0.4992290977   -0.1096717957   -0.0085383726
O    0.5171502824   -1.3144519260   -0.0018164712
O    1.6732075716    0.6412767954    0.0585852155
H    1.9604801740    0.9037879289   -0.8259062765
```

MP2/aVDZ

```
VIp, ENERGY=-283.72852107784
N    2.0148118149    0.0191769213    0.0000000000
H    2.0350684446   -0.6033691152    0.8091570000
H    2.0350684446   -0.6033691152   -0.8091570000
C    0.7619684719    0.7578226821    0.0000000000
H    0.7284546682    1.4171203811    0.8850510000
H    0.7284546682    1.4171203811   -0.8850510000
C   -0.5074942745   -0.0985356087    0.0000000000
O   -0.5193875788   -1.3125367196    0.0000000000
O   -1.6890580540    0.6007315566    0.0000000000
H   -1.5018892407    1.5529034490    0.0000000000
```

```
TS, ENERGY=-283.71664870726
N   -2.0258028832    0.0401605496   -0.0063013502
H   -2.0724835898   -0.5828595582    0.8011193386
H   -2.0626401543   -0.5762784253   -0.8193058273
C   -0.7564025164    0.7513363941    0.0073843094
H   -0.6916046473    1.3929992194    0.9015292977
H   -0.7021522832    1.4378508402   -0.8551456718
C    0.4988439932   -0.1107195949   -0.0093569496
O    0.5140293922   -1.3221482593   -0.0016454994
O    1.6779055445    0.6444833714    0.0594118126
H    1.9551995917    0.8976114724   -0.8339509447
```

CCSD/aVDZ

```
VIp, ENERGY=-283.75729559
N   -2.0162556633   -0.0166424352   -0.0000002455
H   -2.0354958768    0.6058943839    0.8095003957
H   -2.0355014503    0.6058841722   -0.8095071143
C   -0.7622255436   -0.7583852354    0.0000008736
H   -0.7282695181   -1.4183417169    0.8866024720
H   -0.7282693038   -1.4183447283   -0.8865982957
C    0.5100321464    0.0993074903    0.0000003940
O    0.5202315710    1.3065859087    0.0000000437
O    1.6874083676   -0.5976316934   -0.0000006432
H    1.5080703833   -1.5479589482    0.0000003760
```

```
TS, ENERGY=-283.74619979
N   -2.0274502306    0.0354518223   -0.0080364189
H   -2.0749236351   -0.5845487213    0.8020260287
H   -2.0574325552   -0.5846930108   -0.8189034716
C   -0.7582828630    0.7527258053    0.0084810238
H   -0.6983090289    1.3952268447    0.9041143489
H   -0.7041726554    1.4384187943   -0.8568390902
C    0.5014534922   -0.1099658721   -0.0059230663
O    0.5161002499   -1.3148481261   -0.0019690270
O    1.6773149495    0.6403037443    0.0576468671
H    1.9518708691    0.8953017313   -0.8328433953
```

CCSD(T)/aVDZ

```
VIp, ENERGY=-283.78925378
N   -2.0217102156    0.0175154711   -0.0000384353
H   -2.0316958385   -0.6108495614   -0.8084976850
H   -2.0338797385   -0.6064793228    0.8117505172
C   -0.7642368289    0.7620882692   -0.0003102081
H   -0.7306789153    1.4222498594   -0.8892176346
H   -0.7308362503    1.4229334731    0.8880853896
C    0.5100344929   -0.0991662899    0.0000269937
O    0.5196196812   -1.3123115362   -0.0000342778
O    1.6941869958    0.5992789700    0.0001343054
H    1.5094619178    1.5518359111    0.0001981236
```

```
TS, ENERGY=-283.77786049
N    2.0330388527   -0.0375820888    0.0072959136
H    2.0722262161    0.5868500301   -0.8030793648
H    2.0572785095    0.5857315996    0.8192261892
C    0.7595312187   -0.7559843017   -0.0078624384
H    0.6979826064   -1.3993173792   -0.9052057149
H    0.7054948011   -1.4422291333    0.8595027823
C   -0.5015039785    0.1104384271    0.0073487996
O   -0.5146996199    1.3212924252    0.0017400213
O   -1.6839600141   -0.6426993510   -0.0585839921
H   -1.9586952672   -0.8922260623    0.8364565405
```

CCSD(T)-F12b/VDZ-F12

```
VIp, ENERGY=-284.087988102071
C    0.0000000000    0.1006594021   -0.5083251671
C    0.0000000000   -0.7547778345    0.7561835284
N    0.0000000000   -0.0165339157    1.9996994610
O    0.0000000000   -0.5943800244   -1.6741171272
O    0.0000000000    1.2989936181   -0.5144021066
H    0.0000000000   -1.5389539523   -1.4883748905
H   -0.8766435785   -1.4085647853    0.7197945135
H    0.8766435785   -1.4085647853    0.7197945135
H   -0.8049279142    0.5979910432    2.0229133848
H    0.8049279142    0.5979910432    2.0229133848
```

```
TS, ENERGY=-284.076142789544
N   -0.0344337195   -0.0186819490    2.0109622874
H    0.5481407604    0.8059839119    2.0896403928
H    0.6060629724   -0.8031345137    2.0183927855
C   -0.7494672051    0.0132802630    0.7530663923
H   -1.3953669991    0.8941090668    0.7100701674
H   -1.4203298268   -0.8466635516    0.6800281855
C    0.1094702408    0.0169387686   -0.4991708273
O    1.3053558455   -0.0043432792   -0.5095110307
O   -0.6340480430    0.0480452521   -1.6672819477
H   -0.8894680132   -0.8444827404   -1.9156797230
```

Anharmonic Couplings

Vibrational Perturbation Theory of the Second-order plus Resonances (VPT2+K) expression of the anharmonic constants are reported in the following equation for the molecule in its transition state geometry:

$$\begin{aligned}
\chi_{kk} &= \frac{\hbar^2}{16\omega_k^2}\left[f_{kkkk} + \frac{f_{kkF}^2}{\tilde{\omega}_F^2}\left(\frac{8\omega_k^2+3\tilde{\omega}_F^2}{4\omega_k^2+\tilde{\omega}_F^2}\right) - \sum_{m=1}^{F-1}\frac{f_{kkm}^2}{\omega_m^2}\left(2+\frac{\omega_m}{2(2\omega_k+\omega_m)}-\frac{\omega_m}{2(2\omega_k-\omega_m)}\right)\right] \\
\chi_{kk'} &= \frac{\hbar^2}{4\omega_k\omega_{k'}}\left[f_{kkk'k'} + \frac{f_{kkF}f_{k'k'F}}{\tilde{\omega}_F^2} + \frac{2f_{kk'F}^2(\omega_k^2+\omega_{k'}^2+\tilde{\omega}_F^2)}{[(\omega_k+\omega_{k'})^2+\tilde{\omega}_F^2][(\omega_k-\omega_{k'})^2+\tilde{\omega}_F^2]} - \sum_{m=1}^{F-1}\frac{f_{kkm}f_{k'k'm}}{\omega_m^2}\right. \\
&\quad \left. - \sum_{m=1}^{F-1}\frac{f_{kk'm}^2}{2\omega_m}\left(\frac{1}{\omega_k+\omega_{k'}+\omega_m}-\frac{1}{\omega_k+\omega_{k'}-\omega_m}+\frac{1}{\omega_k-\omega_{k'}+\omega_m}-\frac{1}{\omega_k-\omega_{k'}-\omega_m}\right)\right] \\
&\quad + \left(\frac{\omega_k}{\omega_{k'}}+\frac{\omega_{k'}}{\omega_k}\right)\sum_{\alpha}B_\alpha(\zeta_{kk'}^\alpha)^2
\end{aligned} \tag{S1}$$

with $k, k' = 1, 2, \ldots, F-1$, $-\omega_F = \omega'_F = -i\tilde{\omega}_F$ and $\chi_{kF} = -i\tilde{\chi}_{kF}$.

At the equilibrium geometry, the anharmonic constant formula becomes as follows:

$$\chi_{kk} = \frac{\hbar^2}{16\omega_k^2}\left[f_{kkkk} - \sum_{m=1}^{F}\frac{f_{kkm}^2}{\omega_m^2}\left(2 + \frac{\omega_m}{2(2\omega_k + \omega_m)} - \frac{\omega_m}{2(2\omega_k - \omega_m)}\right)\right]$$

$$\chi_{kk'} = \frac{\hbar^2}{4\omega_{k'}\omega_k}\left[f_{kkk'k'} - \sum_{m=1}^{F}\frac{f_{kkm}f_{k'k'm}}{\omega_m^2} + \sum_{m=1}^{F}\frac{2f_{kk'm}^2(\omega_k^2 + \omega_{k'}^2 - \omega_m^2)}{[(\omega_k + \omega_{k'})^2 - \omega_m^2][(\omega_k - \omega_{k'})^2 - \omega_m^2]}\right]$$
$$+ \left(\frac{\omega_k}{\omega_{k'}} + \frac{\omega_{k'}}{\omega_k}\right)\sum_\alpha B_\alpha(\zeta_{kk'}^\alpha)^2$$
$$= \frac{\hbar^2}{4\omega_{k'}\omega_k}\left[f_{kkk'k'} - \sum_{m=1}^{F}\frac{f_{kkm}f_{k'k'm}}{\omega_m^2} - \sum_{m=1}^{F}\frac{f_{kk'm}^2}{2\omega_m}\left(\frac{1}{\omega_k + \omega_{k'} + \omega_m} - \frac{1}{\omega_k + \omega_{k'} - \omega_m}\right.\right.$$
$$\left.\left. + \frac{1}{\omega_k - \omega_{k'} + \omega_m} - \frac{1}{\omega_k - \omega_{k'} - \omega_m}\right)\right] + \left(\frac{\omega_k}{\omega_{k'}} + \frac{\omega_{k'}}{\omega_k}\right)\sum_\alpha B_\alpha(\zeta_{kk'}^\alpha)^2 \tag{S2}$$

where now $k, k' = 1, 2, \ldots, F$ since all frequencies are real. In both the equations B_α is the rotational constant with respect to the α rotational axis, and $\zeta_{kk'}^\alpha$ is the related Coriolis coupling tensor. The same symbol ω for vibrational frequencies and χ anharmonic couplings are used for both equilibrium geometries and TS ones.

Calculated Rates for Glycine

We report for each electronic structure method, the harmonic and anharmonic zero point energy (ZPE) corrected barriers. The harmonic ZPE is computed as

$$ZPE_{harm} = \frac{1}{2}\sum_k \hbar\omega_k$$

where the summation is extended to all the modes for the reactant and all but the reactive mode in the transition state. The anharmonic ZPE is defined accordingly to the perturbative energy expression

$$ZPE_{anharm} = \frac{1}{2}\sum_k \hbar\omega_k + \frac{1}{4}\sum_{k\leq k'}\chi_{k,k'}$$

$-i\omega_F$ is the absolute value of the imaginary frequency of the reactive mode. In the following tables, we report the SCTST rate, the half-life time $t_{1/2}$ computed according to Eq 3 in the main text to account for the two reaction channels, and the harmonic TST result computed as

$$k_{TST}(T) = \frac{1}{\beta h}\frac{Q^{\dagger}_{rot}Q^{\dagger}_{vib}}{Q_{rot}Q_{vib}}e^{-\beta \Delta E_{harm}}$$

where $\beta = \frac{1}{k_B T}$ and both the barrier and the vibrational partition function have been evaluated using the harmonic frequencies.

The evaluation of the Cumulative Reaction Probability (CRP) and the vibrational Density of States (DOS) performed with the Parsctst and Paradensum routines from the Multiwell suite of programs used an energy bin width of 5 cm^{-1}, a total energy window of 30000 cm^{-1}, with a flatness threshold in between 96% and 98%.

B3LYP/aVDZ

Harmonic barrier ($kJ\,mol^{-1}$):	26.69346
Anharmonic barrier ($kJ\,mol^{-1}$):	26.76762
$-i\omega_F$ (cm^{-1}):	560.71208

$T\,(K)$	$k_{TST}\,(s^{-1})$	$k_{SCTST}\,(s^{-1})$	$t_{1/2}(s)$
4	———	2.42837E-02	14.27
6	5.52892E-222	3.71081E-02	9.34
8	9.06823E-164	5.05091E-02	6.86
10	8.09827E-129	6.45294E-02	5.37
12	1.67308E-105	7.92128E-02	4.38
14	7.71763E-89	9.46044E-02	3.66
16	2.47222E-76	1.10750E-01	3.13

MP2/aVDZ

Harmonic barrier ($kJ\,mol^{-1}$): 26.88775
Anharmonic barrier ($kJ\,mol^{-1}$): 26.97315
$-i\omega_F$ (cm^{-1}): 548.57573

$T\,(K)$	$k_{TST}\,(s^{-1})$	$k_{SCTST}\,(s^{-1})$	$t_{1/2}(s)$
4	————	1.08440E-02	31.96
6	1.06900E-223	1.65771E-02	20.91
8	4.70350E-165	2.25735E-02	15.35
10	7.59327E-130	2.88531E-02	12.01
12	2.32800E-106	3.54363E-02	9.78
14	1.42368E-89	4.23449E-02	8.18
16	5.63481E-77	4.96018E-02	6.99

B3LYPD3/aVTZ

Harmonic barrier ($kJ\,mol^{-1}$): 26.52805
Anharmonic barrier ($kJ\,mol^{-1}$): 26.29664
$-i\omega_F$ (cm^{-1}): 559.95017

$T\,(K)$	$k_{TST}\,(s^{-1})$	$k_{SCTST}\,(s^{-1})$	$t_{1/2}(s)$
4	————	4.49059E-02	7.72
6	8.42069E-218	6.86110E-02	5.05
8	1.24320E-160	9.33731E-02	3.71
10	2.61820E-126	1.19270E-01	2.91
12	2.06476E-103	1.46382E-01	2.37
14	4.78757E-87	1.74795E-01	1.98
16	9.15661E-75	2.04600E-01	1.69

CCSD/aVDZ

Harmonic barrier ($kJ\,mol^{-1}$): 24.99555
Anharmonic barrier ($kJ\,mol^{-1}$): 25.09185
$-i\omega_F$ (cm^{-1}): 535.23710

$T\,(K)$	$k_{TST}\,(s^{-1})$	$k_{SCTST}\,(s^{-1})$	$t_{1/2}(s)$
4	3.47019E-262	4.61971E-02	7.50
6	3.25032E-171	7.06507E-02	4.91
8	1.08295E-125	9.62526E-02	3.60
10	2.34508E-98	1.23091E-01	2.82
12	4.05894E-80	1.51258E-01	2.29
14	4.42658E-67	1.80851E-01	1.92
16	2.70381E-57	2.11972E-01	1.63

B2PLYP-D3BJ/aVDZ

Harmonic barrier ($kJ\,mol^{-1}$): 26.90088
Anharmonic barrier ($kJ\,mol^{-1}$): 24.74830
$-i\omega_F$ (cm^{-1}): 557.73560

$T\,(K)$	$k_{TST}\,(s^{-1})$	$k_{SCTST}\,(s^{-1})$	$t_{1/2}(s)$
4	———	2.57983E-01	1.34
6	8.20874E-224	3.94082E-01	0.88
8	3.85735E-165	5.36176E-01	0.65
10	6.47795E-130	6.84703E-01	0.51
12	2.03899E-106	8.40109E-01	0.41
14	1.27055E-89	1.00287E+00	0.35
16	5.09982E-77	1.17349E+00	0.30

CCSD(T)/aVDZ

Harmonic barrier ($kJ\,mol^{-1}$): 25.95747
Anharmonic barrier ($kJ\,mol^{-1}$): 26.03006
$-i\omega_F$ (cm^{-1}): 538.52177

$T\,(K)$	$k_{TST}\,(s^{-1})$	$k_{SCTST}\,(s^{-1})$	$t_{1/2}(s)$
4	———	1.98088E-02	17.50
6	1.34150E-215	3.02893E-02	11.44
8	5.57682E-159	4.12576E-02	8.40
10	5.49024E-125	5.27511E-02	6.57
12	2.60751E-102	6.48076E-02	5.35
14	4.20755E-86	7.74659E-02	4.47
16	6.12857E-74	9.07637E-02	3.82

CCSD(T)-F12b/VDZ-F12

Harmonic barrier ($kJ\,mol^{-1}$): 27.06513
Anharmonic barrier ($kJ\,mol^{-1}$): 27.04525
$-i\omega_F$ (cm^{-1}): 554.8537

$T\,(K)$	$k_{TST}\,(s^{-1})$	$k_{SCTST}\,(s^{-1})$	$t_{1/2}(s)$
4	———	1.02926E-02	33.67
6	3.05061E-225	1.57381E-02	22.02
8	3.26506E-166	2.14369E-02	16.17
10	8.98535E-131	2.74083E-02	12.64
12	3.93108E-107	3.36725E-02	10.29
14	3.09908E-90	4.02511E-02	8.61
16	1.48390E-77	4.71675E-02	7.35

Calculated Rates for Trideuterated Glycine

B3LYPD3/aVTZ

Harmonic barrier ($kJ\,mol^{-1}$): 27.19230
Anharmonic barrier ($kJ\,mol^{-1}$): 27.27976
$-i\omega_F$ (cm^{-1}): 414.11328

$T\,(K)$	$k_{TST}\,(s^{-1})$	$k_{SCTST}\,(s^{-1})$	$t_{1/2}(h)$
4	———	3.46681E-07	278
6	2.38119E-226	5.33757E-07	180
8	4.82032E-167	7.32 774E-07	131
10	1.94438E-131	9.44967E-07	102
12	1.09760E-107	1.17168E-06	82
14	1.03801E-90	1.41439E-06	68
16	5.69661E-78	1.67472E-06	57

CCSD/aVDZ

Harmonic barrier ($kJ\,mol^{-1}$): 25.95044
Anharmonic barrier ($kJ\,mol^{-1}$): 25.42051
$-i\omega_F$ (cm^{-1}): 395.04701

$T\,(K)$	$k_{TST}\,(s^{-1})$	$k_{SCTST}\,(s^{-1})$	$t_{1/2}(h)$
4	———	9.17997E-07	105
6	1.54300E-215	1.41498E-06	68
8	6.19239E-159	1.94513E-06	49
10	5.96882E-125	2.51201E-06	38
12	2.79532E-102	3.11955E-06	31
14	4.46624E-86	3.77204E-06	26
16	6.45882E-74	4.47431E-06	22

CCSD(T)/aVDZ

Harmonic barrier ($kJ\,mol^{-1}$): 26.85443
Anharmonic barrier ($kJ\,mol^{-1}$): 26.89543
$-i\omega_F$ (cm^{-1}): 397.73106

$T\,(K)$	$k_{TST}\,(s^{-1})$	$k_{SCTST}\,(s^{-1})$	$t_{1/2}(h)$
4	———	9.96702E-08	966
6	2.08475E-223	1.53669E-07	626
8	7.76207E-165	2.11305E-07	456
10	1.13350E-129	2.72973E-07	353
12	3.24919E-106	3.39087E-07	284
14	1.89261E-89	4.10081E-07	235
16	7.21584E-77	4.86413E-07	198

CCSD(T)-F12b/VDZ-F12

Harmonic barrier ($kJ\,mol^{-1}$):	27.98077
Anharmonic barrier ($kJ\,mol^{-1}$):	27.83454
$-i\omega_F$ (cm^{-1}):	408.13039

$T\,(K)$	$k_{TST}\,(s^{-1})$	$k_{SCTST}\,(s^{-1})$	$t_{1/2}(h)$
4	———	4.06409E-07	237
6	3.25735E-233	6.24247E-07	154
8	3.42904E-172	8.54695E-07	113
10	1.48078E-135	1.09891E-06	88
12	4.06015E-111	1.35801E-06	71
14	1.18722E-93	1.63299E-06	59
16	1.51897E-80	1.92470E-06	50

Wagner Deep Tunneling Corrected Rates

B3LYPD3/aVTZ (ND)

$T\,(K)$	$k_{SCTST}\,(s^{-1})$	$t_{1/2}(s)$	$k_{SCTST}\,(s^{-1})$ (Wagner)	$t_{1/2}(s)$ (Wagner)
4	4.49059E-02	7.72	4.13956E-02	8.37
6	6.86110E-02	5.05	6.32616E-02	5.48
8	9.33731E-02	3.71	8.61147E-02	4.02
10	1.19270E-01	2.91	1.10028E-01	3.15
12	1.46382E-01	2.37	1.35078E-01	2.57
14	1.74795E-01	1.98	1.61345E-01	2.15
16	2.04600E-01	1.69	1.88916E-01	1.83

B3LYPD3/aVTZ (TD)

$T\,(K)$	$k_{SCTST}\,(s^{-1})$	$t_{1/2}(h)$	$k_{SCTST}\,(s^{-1})$ (Wagner)	$t_{1/2}(h)$ (Wagner)
4	3.46681E-07	278	3.00577E-07	320
6	5.33757E-07	180	4.62958E-07	208
8	7.32774E-07	131	6.35865E-07	151
10	9.44967E-07	102	8.20403E-07	117
12	1.17168E-06	82	1.01778E-06	95
14	1.41439E-06	68	1.22931E-06	78
16	1.67472E-06	57	1.45647E-06	66

Frequencies and Anharmonicities for Glycine

We report the harmonic frequencies and anharmonicity matrices for each level of theory and optimized structure. The frequencies are sorted in increasing order, and for the TS the module of the reactive mode imaginary frequency is reported as the first entry.

B2PLYP/aVDZ

	VIp	TS
1	77.079659	557.735604
2	207.120771	101.310991
3	260.300682	208.181442
4	457.540416	256.191757
5	463.708302	461.555762
6	570.551018	517.566888
7	640.936527	642.424827
8	834.429089	834.392584
9	921.432609	875.240256
10	928.406543	923.009707
11	1124.896019	1074.672983
12	1168.111631	1124.766823
13	1184.367626	1164.358264
14	1289.339419	1218.603934
15	1376.481826	1370.074868
16	1393.188273	1381.861023
17	1456.969702	1441.399804
18	1666.805385	1664.621444
19	1821.745171	1805.480549
20	3048.486140	3061.706942
21	3093.312330	3107.112861
22	3511.258894	3511.697474
23	3589.014867	3590.592612
24	3787.131517	3823.969833

VIp:

	1	2	3	4	5	6
1	2.798389					
2	53.867737	8.426207				
3	-1.204599	2.739721	0.520153			
4	1.130282	-0.802817	-0.624270	-4.758204		
5	-3.392180	2.585110	-0.014760	-1.498034	-0.202626	
6	3.552482	-2.886147	-0.491447	-8.176243	1.793328	-1.262283
7	1.386756	0.814113	-0.163762	0.202535	-0.430679	-1.332428
8	-0.145735	4.276170	-1.851276	-2.466365	-1.805724	-0.739806
9	-0.918551	6.535601	-0.102860	-0.577282	-0.818492	2.524054
10	0.517937	2.064652	-1.496897	1.739521	-1.045010	-1.350674
11	1.088957	1.442371	1.201551	0.918285	-2.695789	-4.254455
12	-1.009487	1.313013	-0.834371	0.599425	-1.118632	0.408321
13	-1.934047	-1.221928	2.152064	-0.205715	0.024299	0.151245
14	0.350764	0.431356	-0.515338	-8.803861	-2.441733	-1.682887
15	-0.114525	1.260805	-3.192139	-0.983249	-1.107885	-0.733948
16	-0.590910	-7.667075	-1.026298	-0.905511	0.801235	-0.934304
17	0.178701	2.668492	-0.450700	-0.426869	0.050319	-3.762718
18	2.591797	4.157605	0.192154	-0.055058	-0.033905	0.205417
19	-0.741919	-0.734617	-0.731366	0.327190	-0.941319	-2.874543
20	0.892069	-1.689662	0.058740	1.006376	0.552328	-1.392563
21	0.682490	-3.871813	0.202604	0.940096	0.808439	-1.317304
22	-9.280309	-1.753966	0.702484	0.290026	-0.075506	0.611471
23	-1.858448	-6.437948	0.836440	0.385678	0.008363	0.681614
24	1.742546	0.094473	0.412847	-4.719523	-0.090204	-3.518076

	7	8	9	10	11	12
7	-0.402743					
8	-0.289567	-2.199146				
9	-0.059142	-1.817041	0.708255			
10	-3.378743	-18.069568	-2.768216	-13.120016		
11	-2.740573	-2.812953	-1.686553	-3.520466	-3.843283	
12	-1.205686	-3.794983	-0.909972	-11.897096	-7.045049	-3.872537
13	-0.147896	-2.011912	-0.206602	-3.223009	-2.247754	-5.120744
14	0.293389	-3.649259	-2.350139	-1.760386	-10.631096	-4.072186
15	-2.600180	-5.197372	-0.553094	-3.864046	-7.564298	-4.278684
16	-0.312936	-2.601695	-1.951528	-5.905262	-2.158008	-4.562246
17	-0.354376	-0.893212	-7.792888	-1.212528	-1.386649	-1.024495
18	1.930524	-2.602554	-1.917833	-12.308629	-1.187961	-2.881857
19	-4.920728	-0.783205	-3.147516	0.654020	-4.954309	-1.428711
20	-0.071232	-0.399822	-4.462958	-1.060535	1.183527	-0.395664
21	0.154818	0.230788	-4.506584	-0.625528	1.027014	0.034402
22	-0.187818	2.100226	-1.239354	8.264914	-0.690286	-2.193611
23	-0.265958	1.789267	-0.563619	8.018909	-0.297488	-1.442140
24	-0.862021	-0.157857	0.231101	-0.069488	-5.855048	-2.517225

	13	14	15	16	17	18
13	-1.228087					
14	-1.732404	-9.375774				
15	-7.233858	-5.802006	-7.799318			
16	-6.479567	-0.793868	-8.272280	-2.712537		
17	-1.274928	-0.278997	-1.494239	-5.059808	-1.370034	
18	-4.367154	-0.324411	-1.072141	-10.575431	0.142410	-2.039451
19	-1.104977	-4.716480	-2.115323	-0.320153	-0.831750	0.030730
20	-6.937097	-0.079325	11.156027	-5.477691	-31.353174	-1.037434
21	-5.240122	-1.719191	-3.444336	-1.959452	-22.450400	0.458653
22	-3.488218	-0.119449	-0.688965	-4.185323	-0.671206	-36.213987
23	-2.745783	-0.054927	-0.517546	-2.156178	-0.434003	-24.016280
24	-0.264362	-8.367698	-0.756110	-0.044772	0.068827	-0.029578

	19	20	21	22	23	24
19	-10.003725					
20	-1.049383	-29.922396				
21	-1.029241	-130.100044	-35.192334			
22	-0.122249	-0.800302	-0.782899	-39.239137		
23	-0.321327	-0.538089	-0.737822	-155.041033	-46.643318	
24	-3.459782	-0.329529	-0.255741	-0.033554	0.002206	-88.513232

TS:

	1	2	3	4	5	6
1	-14.625679					
2	-3.108046	2.720970				
3	-0.131234	33.344677	1.319072			
4	0.486055	0.507547	3.666744	0.626188		
5	1.220605	0.618621	2.675757	0.085268	-0.161215	
6	1.477982	0.072406	-4.372078	-0.258314	-1.978711	0.769187
7	1.026343	-0.152022	0.251356	-0.226373	-0.680139	-0.736777
8	1.960241	-0.403099	3.779896	-1.261133	-2.532587	-1.770210
9	1.881999	0.775888	6.852134	-0.067924	-0.209112	-2.177743
10	1.301388	-0.458354	-1.277401	-0.886996	-1.258869	-2.845924
11	-3.973099	1.287625	0.983512	-0.624217	-0.191550	0.923574
12	3.453219	0.271516	-0.549425	-0.869846	-2.308675	-1.362320
13	2.202076	-0.716934	0.404454	-0.561099	-1.621999	0.099282
14	-5.622842	-1.492049	0.305319	0.571983	-0.064427	-1.916329
15	0.698846	-0.421080	-0.064321	-1.143424	-0.916562	-0.746196
16	0.581684	-0.922240	-5.753593	0.241537	0.332527	-0.942296
17	1.022761	1.911382	4.107395	-0.627506	-0.073717	1.394404
18	0.170143	2.387743	4.023346	0.218743	-0.017422	0.352646
19	-1.624400	-0.599236	-0.959165	-0.557134	-0.926829	-1.816789
20	-0.592587	1.555458	-1.823326	0.241323	0.737772	-1.455164
21	0.045024	-0.790826	-2.832326	0.390762	0.879809	-1.263893
22	0.001359	-5.393405	-0.615451	0.594330	-0.091081	0.816510
23	-0.107980	-1.871505	-2.670167	0.714068	-0.010093	0.874181
24	10.425657	-0.271643	-0.036858	0.040449	-0.365925	-0.428318

	7	8	9	10	11	12
7	-0.135806					
8	-1.034298	-2.657920				
9	0.593461	-1.261133	0.159197			
10	-3.504441	-22.407400	-2.140904	-11.921827		
11	-0.525214	-1.178033	-1.482929	-2.039018	-4.782607	
12	-2.793312	-4.694545	-1.147507	-6.975783	-3.947553	-3.076251
13	-1.580789	-4.833136	-0.478766	-9.741940	-4.168275	-8.320098
14	-0.304027	-1.738281	-2.784909	-2.340183	-11.871763	-3.215159
15	-2.181027	-3.919489	-0.357572	-3.736619	-2.535893	-4.628353
16	-0.391892	-3.007655	-1.755807	-5.083474	-1.895174	-3.661238
17	-0.400173	-0.987729	-3.967249	-0.866711	0.067456	-1.233249
18	1.926003	-3.339363	-1.846600	-11.005846	-1.513641	-2.069052
19	-4.774691	0.405953	-1.272447	0.702450	-1.518783	-1.847945
20	0.116654	-0.517540	-4.789176	-0.844598	-0.578208	-0.232047
21	0.310266	0.201870	-5.224570	-0.306527	-1.420197	1.121688
22	-0.133326	2.961302	-0.700615	8.071855	-1.283431	-1.382126
23	-0.179858	2.834767	-0.228345	8.672417	-0.878027	-0.870540
24	-0.546451	-0.527970	-2.943348	-0.161248	-8.539307	-4.075503

	13	14	15	16	17	18
13	-3.651150					
14	-3.174901	-2.098529				
15	-5.571184	-6.770164	-7.839702			
16	-3.678517	-3.559714	-7.972067	-3.042535		
17	-0.482619	-0.346300	-1.696047	-3.217011	-7.016874	
18	-2.513273	-2.624518	-5.882163	-9.942909	0.132964	-2.044571
19	-1.108493	-1.486689	-1.894386	-0.253149	-0.881279	0.031923
20	-1.459918	-4.452554	13.735608	-5.749189	-11.198000	3.853752
21	-1.164256	-4.183552	-5.373314	-3.243985	-23.160790	-0.165361
22	-2.028237	-1.928011	-1.080036	-4.036910	-0.558290	-36.335055
23	-1.236319	-1.467181	-0.852655	-2.176493	-0.308943	-23.790862
24	-0.882524	-6.904309	-0.519029	-0.234344	0.075868	-0.018127

	19	20	21	22	23	24
19	-10.991307					
20	-1.441231	-36.287970				
21	-1.513794	-105.701167	-39.701776			
22	-0.089598	-0.762623	-0.836559	-39.587676		
23	-0.312054	-0.540582	-0.793051	-155.318529	-46.425456	
24	-1.285690	0.423784	0.248972	-0.067662	-0.084749	-86.984419

B3LYP/aVDZ

	VIp	TS
1	76.892442	560.712077
2	199.930271	100.679258
3	258.791225	200.686650
4	458.129543	255.115261
5	460.719379	458.980948
6	570.699881	517.485700
7	641.860247	643.354036
8	825.851369	826.873590
9	909.218123	868.802124
10	916.787682	906.262394
11	1118.340485	1063.706783
12	1158.615644	1116.643819
13	1177.322258	1154.785226
14	1282.816168	1209.924182
15	1364.167935	1358.417280
16	1382.169166	1370.907468
17	1443.300111	1427.086699
18	1658.632046	1656.419483
19	1836.608196	1824.659352
20	3021.573471	3034.089933
21	3060.608853	3073.949363
22	3496.197427	3496.577337
23	3568.634820	3570.044949
24	3773.567066	3809.964541

VIp:

	1	2	3	4	5	6
1	3.135849					
2	52.949732	10.705045				
3	0.866621	4.038030	0.492194			
4	1.513940	-0.367275	-0.511189	-4.087008		
5	-3.085645	2.914334	-0.023383	-1.471050	0.059818	
6	3.253399	-2.595479	-0.524660	-9.726256	1.459262	-1.795362
7	1.251749	0.665320	-0.712597	0.050104	-0.546101	-1.279280
8	-0.212198	4.552428	-1.772200	-2.210558	-1.550879	-0.667074
9	0.816921	3.243693	-0.484961	-1.280480	-2.400056	-1.294194
10	-1.094648	6.641415	-0.096110	0.772985	-1.380043	1.792196
11	1.059429	1.792960	-0.027828	1.163402	-2.720461	-2.828063
12	-1.342290	0.952735	-0.988141	0.742052	-1.176165	0.461491
13	-2.593100	-1.660675	1.744182	-0.281355	-0.112062	-0.086012
14	0.174844	0.525969	-0.695257	-9.076858	-2.800099	-2.445157
15	-0.118888	0.975058	-2.125006	-2.783552	-2.559883	-0.885671
16	-0.941539	-7.502907	-0.933271	-0.302012	1.299027	-0.946814
17	-0.632836	2.712236	-0.498488	-0.348207	0.083854	-3.675305
18	0.909693	2.837314	0.133404	-0.061223	-0.052227	0.151063
19	-0.826683	-0.925020	-0.614016	-0.632838	0.632397	-2.757299
20	1.071114	-2.041368	0.079127	0.923761	0.564919	-1.371459
21	0.887843	-4.759044	0.213406	0.847967	0.843506	-1.149867
22	-8.960415	-2.467597	0.545711	0.300815	-0.061756	0.612393
23	-2.073233	-6.585621	0.603031	0.411787	0.005764	0.696073
24	1.465312	0.051414	0.344503	-2.229860	-0.245744	-3.045276

	7	8	9	10	11	12
7	-0.073654					
8	-0.466743	-2.266319				
9	-2.899986	-18.677262	-11.890967			
10	-0.070887	-2.020501	-2.721927	0.290330		
11	-2.623625	-2.357857	-3.538864	-1.440697	-3.798913	
12	-1.278580	-3.968004	-10.606128	-1.016253	-7.721658	-3.728357
13	-0.249578	-2.331982	-3.716580	-0.425903	-2.554452	-4.987687
14	-1.216126	-3.976302	-1.927982	-2.166815	-10.654029	-4.412797
15	-2.481382	-4.627787	-3.480410	-2.282991	-6.208729	-3.991943
16	-0.362253	-2.874691	-5.585775	-1.440421	-2.249606	-4.370128
17	-0.391070	-0.995392	-1.129766	-7.590676	-1.546327	-1.030193
18	1.875229	-2.613659	-11.596096	-2.177567	-1.262563	-2.725474
19	-4.597432	-1.227249	1.658409	-1.760004	-4.660351	-1.610231
20	-0.068308	-0.596044	-1.068699	-4.078794	1.509011	-0.197589
21	0.167151	0.023130	-0.563404	-3.811791	1.377513	0.172322
22	-0.144219	2.693387	8.504785	-1.148390	-0.744027	-2.072552
23	-0.268356	2.212663	7.991196	-0.361350	-0.318007	-1.334691
24	-0.963509	-0.244867	-0.079506	0.144063	-4.846339	-2.376785

	13	14	15	16	17	18
13	-1.372746					
14	-1.720411	-9.347223				
15	-7.912857	-5.987798	-8.011725			
16	-6.600710	-0.899011	-8.321273	-2.807420		
17	-1.290038	-0.226035	-1.104954	-5.739889	-1.024314	
18	-4.776965	-0.343691	-1.002553	-12.009151	0.108761	-1.455060
19	-1.067079	-3.997617	-0.307273	-0.965641	-0.746080	-0.011104
20	-6.089452	0.252652	13.183187	-4.744285	-31.217497	-1.043418
21	-4.030591	-1.523465	-2.086882	0.360635	-21.021824	1.511686
22	-3.033867	-0.112929	-0.763676	-3.272934	-0.780085	-35.715578
23	-2.112059	-0.032533	-0.585875	-0.934981	-0.523162	-22.025818
24	-0.320763	-4.770465	-0.447446	-0.150756	-0.000879	-0.059375

	19	20	21	22	23	24
19	-9.641384					
20	-1.094459	-30.809567				
21	-1.113304	-134.678657	-36.025418			
22	-0.079316	-0.755247	-0.791217	-39.719254		
23	-0.351791	-0.523339	-0.790694	-157.194287	-47.032265	
24	-3.369339	-0.300257	-0.232494	0.001980	0.060997	-87.042628

TS:

	1	2	3	4	5	6
1	-13.878707					
2	-3.640898	3.890389				
3	-0.002616	47.308713	6.597678			
4	0.486502	-0.030358	2.881166	0.575935		
5	1.151972	0.667556	2.991896	0.138929	0.242562	
6	0.600285	-0.223644	-4.149368	-0.263223	-2.088237	0.539454
7	0.703983	-0.301558	0.228753	-0.758562	-0.838573	-0.747335
8	1.399967	-0.608996	4.871175	-1.105093	-2.262889	-1.715487
9	0.610167	0.085032	6.585757	-0.122036	-0.274833	-3.449429
10	1.010515	-0.380517	0.508271	0.094282	-4.379423	-2.551978
11	-6.249121	0.573609	1.107230	-0.614701	-0.338298	0.133878
12	2.645257	-0.005072	0.554084	-1.448314	-2.235396	-1.467242
13	2.244149	-1.112795	0.366064	-0.755238	-1.707865	-0.145046
14	-6.131567	-2.158767	0.680779	0.398130	-0.214316	-2.128431
15	0.564522	-0.736014	-1.077314	-0.875892	-3.586392	-1.028429
16	0.601843	-1.561575	-6.610757	0.054771	1.467810	-0.835717
17	1.314285	0.952244	3.447204	-0.641404	-0.049869	0.909649
18	0.187797	1.074413	2.412414	0.116627	-0.036571	0.277042
19	-1.065760	-0.671070	-1.247829	-0.469808	1.814354	-1.788127
20	-0.610212	1.497835	-2.254600	0.240847	0.805418	-1.288479
21	0.294106	-0.306471	-3.386268	0.448599	0.915256	-0.767252
22	-0.003701	-7.881913	-2.134321	0.444850	-0.089114	0.806063
23	-0.104564	-1.646490	-4.058617	0.495873	-0.016117	0.921705
24	14.555843	-0.080726	0.012374	0.003660	-0.421162	-0.114202

	7	8	9	10	11	12
7	-0.157292					
8	-1.213092	-3.105530				
9	0.551085	-1.443775	-0.108624			
10	-2.947180	-23.191190	-2.044358	-10.487299		
11	-0.659295	-1.247664	-2.712370	-2.047560	-5.197816	
12	-2.638962	-4.477291	-1.387677	-5.874208	-4.182114	-3.023489
13	-1.746876	-5.165452	-0.657638	-8.652706	-4.090391	-8.565323
14	-0.415090	-1.963359	-3.315046	-2.626641	-11.901475	-3.545070
15	-2.047071	-3.401521	-0.353797	-3.406205	-2.858290	-4.192433
16	-0.444965	-3.458339	-1.830492	-3.517261	-2.149323	-3.868577
17	-0.434554	-1.136373	-4.029520	-0.875849	-0.047379	-1.412027
18	1.838192	-3.619488	-2.178007	-8.988067	-1.674408	-2.148838
19	-4.248022	0.161010	-1.038829	2.650270	-1.340949	-1.571738
20	0.119808	-0.820158	-4.223697	-0.732307	-0.212198	0.266912
21	0.318530	-0.117505	-4.340294	-0.157923	-0.959251	1.500663
22	-0.068383	4.022785	-0.558587	7.884804	-1.095639	-1.310333
23	-0.167435	3.497519	0.034207	7.913691	-0.599256	-0.765701
24	-0.482676	-0.454354	-2.135004	-0.206235	-4.997669	-3.526891

	13	14	15	16	17	18
13	-3.587616					
14	-3.226706	-2.093451				
15	-5.317693	-7.713141	-8.294700			
16	-3.944128	-3.796584	-8.508401	-3.222587		
17	-0.514548	-0.914242	-1.565387	-3.857126	-7.206198	
18	-2.460355	-3.012197	-1.704194	-11.924909	0.114488	-1.503749
19	-0.932335	-1.476106	0.970987	-0.391392	-0.739249	0.069761
20	-0.964444	-4.219541	11.903372	-3.300871	-9.293997	0.987380
21	-0.898084	-3.197305	-3.253724	-1.879325	-21.308184	0.067086
22	-1.944383	-1.646250	-1.098539	-3.108857	-0.667213	-35.815055
23	-1.161524	-1.039832	-0.873671	-0.955378	-0.410533	-21.928944
24	-0.908829	-4.798793	-0.494431	-0.199354	0.042179	-0.019577

	19	20	21	22	23	24
19	-10.568023					
20	-1.571867	-39.553909				
21	-1.528034	-101.574620	-42.857267			
22	-0.107675	-0.713334	-0.879136	-39.972354		
23	-0.341631	-0.521606	-0.849742	-157.733610	-47.337643	
24	-1.427850	0.497673	0.291336	-0.055067	-0.071130	-85.774385

B3LYPD3/aVTZ

	VIp	TS
1	82.347450	559.950169
2	200.770255	102.539081
3	264.550995	201.584740
4	464.644825	259.088440
5	465.415971	463.093802
6	571.028702	519.968008
7	647.993970	649.604580
8	824.290528	828.392598
9	910.629489	878.315985
10	928.263741	907.752448
11	1120.298602	1064.238263
12	1154.784170	1119.310357
13	1190.923329	1150.685200
14	1283.185292	1220.853256
15	1377.044546	1373.131798
16	1395.551169	1383.483351
17	1465.815913	1448.546315
18	1679.408141	1677.353165
19	1843.780976	1832.481893
20	3011.882861	3024.467974
21	3043.979040	3058.633510
22	3502.842943	3503.214177
23	3570.403002	3571.818409
24	3777.062627	3815.118356

VIp:

	1	2	3	4	5	6
1	3.455161					
2	59.844801	12.627752				
3	0.010869	6.606823	0.639318			
4	-0.116676	-0.454076	-0.807526	-4.951158		
5	-4.097648	2.677347	-0.388861	-1.966367	-0.157689	
6	3.404486	-2.647289	-0.603172	-9.458540	1.743395	-1.721201
7	1.163946	-0.045679	0.016003	-0.183546	-0.844358	-1.311068
8	0.280038	4.496984	-1.714926	-2.435337	-2.642407	-0.717749
9	1.004986	3.336761	-1.066977	-3.368873	-2.120962	-1.300371
10	-1.337088	4.012015	0.023125	-0.481914	-0.459445	1.477291
11	0.535618	1.009900	-0.342300	0.997876	-2.494181	-2.739623
12	-0.746134	2.424015	-0.732690	0.910960	-1.155399	1.566026
13	-3.100584	-1.984379	1.653279	-0.345817	-0.447620	-0.141126
14	-1.126679	0.341728	-1.434003	-10.241202	-2.672646	-3.373118
15	-0.289326	0.987915	-1.869622	-1.353792	-1.452434	-0.646776
16	-1.212804	-7.395090	-0.921109	-0.995561	-0.261137	-0.824558
17	-1.346919	1.771145	-0.481631	-1.042977	-0.143842	-3.545429
18	0.855066	1.617966	0.006808	-0.139632	-0.131610	-0.076359
19	-0.853776	-0.913613	-0.614523	-0.052824	-0.129727	-2.683290
20	0.937677	-1.960172	0.051461	1.004201	0.596251	-1.191771
21	1.135033	-4.172138	0.222336	0.963324	0.882330	-0.850012
22	-9.012145	-2.337748	0.634176	0.280590	-0.076705	0.637536
23	-2.212769	-5.929582	0.796458	0.398255	0.003797	0.723077
24	1.462693	0.100494	0.373540	-2.376687	-0.210687	-2.809803

	7	8	9	10	11	12
7	0.102682					
8	-0.283815	-2.093202				
9	-3.647978	-15.620067	-11.383120			
10	-0.256223	-2.119843	-3.044962	0.734202		
11	-2.550540	-2.380226	-5.399980	-1.812764	-3.583651	
12	-1.271539	-3.597665	-12.185280	-0.341565	-8.859175	-3.361041
13	-0.358673	-2.468354	-4.251454	-1.234289	-2.951391	-5.438158
14	-2.258997	-3.728996	-1.846050	-2.436580	-10.440966	-5.494546
15	-2.175986	-4.275625	-3.333676	-1.479875	-5.276679	-3.642979
16	-0.467044	-2.297908	-6.171217	-2.908604	-2.885126	-4.117082
17	-0.592837	-1.059233	-1.068372	-8.027060	-1.745963	-1.174952
18	1.491318	-2.841156	-18.284989	-2.897446	-1.605592	-3.030090
19	-4.542404	-1.462460	1.254281	-5.270991	-4.400804	-2.102231
20	-0.051078	-0.428459	-1.084146	-3.745466	1.549322	-0.017820
21	0.188685	0.200744	-0.504450	-3.081805	1.507648	0.353575
22	-0.191020	2.152771	8.913256	-0.955805	-0.834491	-1.660906
23	-0.294480	1.748164	8.055673	-0.216824	-0.398358	-0.965252
24	-0.953100	-0.153154	-0.112415	0.175384	-4.121048	-2.831233

	13	14	15	16	17	18
13	-1.610747					
14	-1.783070	-10.977311				
15	-9.446420	-3.495692	-10.064950			
16	-7.454681	-1.373163	-10.282976	-3.169956		
17	-1.971615	-0.646802	-1.993999	-7.738909	-1.626429	
18	-5.511380	-0.248262	1.247681	-8.348536	-0.278718	-2.572002
19	-1.311455	-4.245829	-0.901830	-0.472585	-0.734207	0.090524
20	-5.612782	0.187413	14.502942	-3.471533	-30.969406	-3.420075
21	-3.027137	-0.508361	-2.451012	-1.649132	-18.789950	-2.484629
22	-2.828446	-0.039306	-0.746645	-2.962114	-0.834352	-35.801905
23	-1.803919	0.053150	-0.587644	-0.549109	-0.621728	-20.412136
24	-0.366615	-3.204033	-0.699389	-0.174334	0.020464	-0.055881

	19	20	21	22	23	24
19	-9.747431					
20	-1.126545	-29.035176				
21	-0.898730	-127.475516	-34.167191			
22	-0.178793	-0.628507	-0.691980	-38.561770		
23	-0.365357	-0.455725	-0.733825	-153.361659	-45.842151	
24	-3.376738	-0.337643	-0.271453	0.006738	0.057402	-84.083798

TS:

	1	2	3	4	5	6
1	-14.535623					
2	-3.309353	4.429747				
3	0.105937	52.891895	7.775703			
4	0.712799	1.939956	6.708474	0.801124		
5	1.228873	0.580126	2.952600	0.059441	0.101477	
6	1.137532	-5.329508	-4.284470	-0.170073	-1.659631	0.640701
7	0.712140	4.789558	-0.184961	0.088796	-1.163147	4.207203
8	1.246742	-0.188428	4.716896	-1.001105	-2.516087	-1.728445
9	0.662323	-0.320841	4.827801	-0.293236	-0.351862	-2.795182
10	1.024154	-0.023909	0.524224	-0.414466	-3.362415	-2.372654
11	-5.306255	0.362228	0.684610	-0.860478	-0.858649	-0.267117
12	1.070794	-0.326656	-0.077504	-1.665664	-1.829555	-1.139869
13	2.494273	-0.294052	0.170784	-0.126974	-1.763967	-0.441553
14	-5.552358	-2.412014	1.865624	0.624887	-0.254632	-1.970295
15	0.744886	-0.610921	-1.181901	-0.559512	-1.313161	-0.890391
16	0.970863	-1.696223	-6.653349	0.002005	0.843018	-0.721401
17	1.248582	0.577745	2.203831	-0.577771	0.008434	-0.174213
18	0.186644	0.968842	1.192128	0.017673	-0.127347	-0.034344
19	-1.222994	-0.717506	-1.227388	-0.469241	-0.107603	-1.637759
20	-0.680916	0.859179	-2.334448	0.194525	0.814239	-1.262895
21	0.271541	-0.096347	-2.835646	0.427886	0.850887	-0.519039
22	-0.019726	-8.068206	-2.175937	0.474650	-0.114658	0.823646
23	-0.117667	-1.905239	-3.696191	0.588146	-0.039156	0.950784
24	14.181167	-0.160575	0.005628	-0.023075	-0.508273	-0.160865

	7	8	9	10	11	12
7	-0.221016					
8	-1.266349	-2.646405				
9	0.343314	-1.099781	-0.670382			
10	-3.723212	-20.676198	-2.315401	-10.023889		
11	-0.978210	-1.805157	-4.003729	-2.511416	-5.251421	
12	-2.152273	-4.607896	-1.887531	-7.834258	-5.816032	-2.898710
13	-1.753230	-5.109563	-0.987152	-9.296578	-4.775907	-8.374365
14	-0.495341	-2.141424	-3.846887	-3.023712	-11.384442	-4.469849
15	-1.851286	-3.299798	-0.839283	-3.317461	-2.932390	-3.714791
16	-0.510150	-3.214889	-2.046697	-4.673510	-2.048296	-4.642796
17	-0.366600	-0.972062	-5.090513	-0.900208	-0.523968	-1.453980
18	1.442297	-4.720366	-3.378600	-14.206107	-1.793083	-2.783514
19	-4.164245	0.096098	-0.923243	1.833701	-1.862688	-1.209538
20	0.128515	-0.760582	-3.995428	-0.738158	0.162198	0.239787
21	0.309995	0.063108	-3.822781	-0.146399	-0.784331	1.656879
22	-0.121276	3.412522	-0.396123	8.156670	-0.820434	-1.410066
23	-0.204487	2.897583	0.143599	7.897311	-0.357521	-0.842859
24	-0.456541	-0.432248	-2.231562	-0.187923	-3.439806	-4.163619

	13	14	15	16	17	18
13	-3.477459					
14	-3.943082	-2.088990				
15	-4.784286	-9.662028	-9.862941			
16	-4.182331	-4.380980	-10.045580	-3.499223		
17	-0.709812	-1.806962	-1.961166	-4.770119	-2.825418	
18	-2.806565	-3.612546	0.012264	-9.026510	-0.243272	-2.624451
19	-1.281071	-2.471412	-0.986629	-0.398676	-0.736642	0.149312
20	-0.588513	-4.110769	10.474577	-4.693007	-30.062101	-5.136703
21	-0.369592	-2.091671	-1.007121	-1.681526	-19.013930	0.997678
22	-1.627886	-1.639276	-1.020459	-2.843148	-0.714816	-35.938265
23	-0.865969	-0.946187	-0.815320	-0.611811	-0.507954	-20.398901
24	-1.551362	-4.209273	-0.447505	-0.233700	0.023238	-0.020884

	19	20	21	22	23	24
19	-10.713113					
20	-1.564504	-39.847960				
21	-0.703793	-86.725949	-42.641685			
22	-0.187442	-0.583823	-0.784696	-38.823991		
23	-0.362395	-0.450416	-0.799250	-153.901705	-46.151405	
24	-1.476516	0.466417	0.285089	-0.063894	-0.080687	-83.276344

CCSD/aVDZ

	VIp	TS
1	80.296399	535.237100
2	218.166005	101.780189
3	261.466398	218.734341
4	446.091966	256.847602
5	466.833511	463.703197
6	570.382022	521.373695
7	647.333703	649.306540
8	850.116709	848.652264
9	926.151647	884.154341
10	955.876655	949.116832
11	1145.555653	1103.301429
12	1180.746034	1141.938723
13	1189.738789	1180.659633
14	1316.130731	1237.767393
15	1399.967698	1387.532088
16	1400.791831	1391.537365
17	1466.094680	1453.502952
18	1674.653248	1672.591314
19	1861.420021	1845.147311
20	3042.692773	3056.004600
21	3087.780916	3102.920118
22	3501.725186	3501.548505
23	3578.357858	3579.349931
24	3819.168557	3848.505005

VIp:

	1	2	3	4	5	6
1	2.036350					
2	41.617711	6.870877				
3	0.646250	4.492063	0.619214			
4	1.955974	-0.152122	-0.654906	-1.895223		
5	-3.987145	2.429275	0.118881	-1.529445	-0.176800	
6	4.341664	-2.941219	-0.522020	-6.703620	2.531607	-1.017612
7	1.093150	0.484439	-0.031937	-0.071245	-0.351132	-1.345982
8	0.877463	2.722249	-1.678495	-2.337998	-2.251513	-1.065289
9	-2.275445	5.735664	-0.072165	0.262407	-0.869561	2.963712
10	-0.116907	0.308242	1.970231	-0.745871	3.738262	-1.460823
11	-0.075098	1.466052	-1.594483	-1.212932	-2.135760	-1.708801
12	-0.669484	1.374116	-3.612851	0.082560	-1.390459	0.086149
13	-1.426089	-0.445165	1.954717	-0.189662	0.035068	0.081032
14	-1.509088	0.098822	-0.390025	-12.358979	-1.443413	-0.990487
15	0.923215	-6.820035	-0.949484	-1.361615	0.774689	-0.868976
16	-0.582824	0.569414	-0.987048	-1.247324	-4.284430	-1.262989
17	-0.703068	2.483204	-0.319193	-0.766700	-0.017883	-4.774256
18	0.712296	2.938804	0.269889	-0.127553	-0.029315	0.168637
19	-0.761077	-0.601828	-0.608788	-0.023067	-2.536928	-2.986356
20	0.535689	-1.512932	-0.036822	0.933381	0.427846	-1.525300
21	0.985096	-3.232779	0.042800	0.844875	0.603280	-1.282522
22	-7.320414	-1.663620	0.670237	0.137281	-0.095244	0.588731
23	-1.302772	-4.990326	0.786588	0.261077	-0.027518	0.653855
24	1.698328	0.013085	0.477433	-1.768518	0.115585	-2.403760

	7	8	9	10	11	12
7	-0.133554					
8	0.127107	-1.814637				
9	-0.007389	-2.857189	-0.426677			
10	-3.395847	-6.399182	-2.567175	-10.989403		
11	-2.181613	-2.713949	-1.319103	-5.658189	-2.998328	
12	-1.498336	-3.325129	-0.427767	-13.006513	-9.007723	-2.713550
13	-0.178670	-1.937261	-0.263038	-2.160429	-2.591675	-4.386902
14	-0.856520	-3.049440	-3.210263	-1.639206	-7.468878	-5.653714
15	-0.304528	-2.265557	-2.028103	-5.593593	-3.062126	-3.559248
16	-2.982979	-5.566790	-0.372969	-7.851643	-5.334807	-5.141102
17	-0.297329	-0.839767	-8.981016	-1.121742	-1.628652	-0.404509
18	2.211618	-9.199368	-2.016589	-16.333365	-1.876091	-1.929756
19	-4.322573	-0.686985	1.508651	0.655944	-3.345386	-2.508673
20	-0.085942	-0.358788	-4.437562	-1.074235	1.402421	-1.084459
21	0.095010	0.098952	-4.059662	-0.781660	1.283358	-1.331344
22	-0.263004	1.686463	-1.179434	7.891155	-1.411505	-1.374018
23	-0.346289	1.138520	-0.492581	6.379883	-1.014457	-0.951741
24	-0.886107	0.019613	0.190512	-0.032612	-3.397827	-3.484736

	13	14	15	16	17	18
13	-1.212169					
14	-2.362684	-8.465331				
15	-6.036403	0.420058	-2.715060			
16	-7.535159	-9.273501	-8.229380	-8.230399		
17	-1.360436	-0.409235	-5.056859	-1.778522	-1.684491	
18	-4.270971	-0.683401	-9.200501	2.345710	0.107143	-2.071408
19	-1.402915	-4.148744	-0.061612	-3.390968	-0.606189	0.026019
20	-6.538695	0.970607	-4.935555	11.544868	-31.381390	-3.932666
21	-4.124356	-2.208327	-2.282654	-0.664754	-20.895744	-0.903594
22	-3.174863	-0.183261	-4.187465	-0.675443	-0.499847	-35.922173
23	-2.364455	-0.128106	-1.885507	-0.551570	-0.294789	-23.543465
24	-0.253140	-5.976514	-0.038564	-0.497135	0.009034	-0.047874

	19	20	21	22	23	24
19	-9.213715					
20	-0.777464	-30.173553				
21	-0.558970	-130.065000	-35.117889			
22	-0.143958	-0.607651	-0.635175	-39.837816		
23	-0.277346	-0.396002	-0.587116	-158.076938	-46.955477	
24	-3.484723	-0.252387	-0.175088	-0.028450	-0.018869	-88.130129

TS:

	1	2	3	4	5	6
1	-14.053381					
2	-3.601590	2.451969				
3	-0.008309	39.302977	4.504844			
4	0.508943	1.322355	4.524552	0.652121		
5	1.108534	0.745491	2.586450	0.248818	-0.130457	
6	0.830612	-5.055751	-3.947693	-0.248824	-1.976636	0.642599
7	0.639612	4.835057	-0.185424	-0.056480	-0.472039	4.285114
8	1.508412	-0.550916	2.939950	-1.025540	-2.454605	-1.898578
9	1.109295	0.046289	7.098052	-0.214675	-0.261851	-2.244640
10	1.024226	-1.177539	-0.008763	2.621348	4.542730	-2.398580
11	-5.861097	0.165614	-0.204490	-1.226665	0.189059	0.733026
12	2.341484	-0.564475	-0.217267	-1.947225	-1.881903	-0.967166
13	3.187599	-0.147738	2.921974	-3.457625	-2.023753	-0.572559
14	-8.500876	-1.626296	0.735039	0.257699	-0.094301	-2.208880
15	0.860432	-0.941398	-3.454563	1.129514	-0.788189	-0.831952
16	0.551920	-1.108126	-4.208320	-0.673323	-3.507436	-1.260371
17	0.907585	0.866714	2.227778	-0.548672	-0.127637	-0.016711
18	0.186971	1.021472	2.723679	0.211209	0.000722	0.236556
19	-0.147975	-0.582440	-0.639687	-0.459404	-2.538704	-2.091319
20	-0.652103	1.460871	-1.420907	0.183240	0.659245	-1.334213
21	-0.103622	0.098961	-2.336942	0.256398	0.696007	-0.837011
22	0.046952	-6.572778	-1.351657	0.605997	-0.100071	0.727353
23	-0.029514	-0.972902	-3.251010	0.717569	-0.039607	0.855955
24	14.827132	-0.023337	0.022568	-0.013789	-0.424171	-0.145728

	7	8	9	10	11	12
7	-0.061531					
8	-0.429788	-1.915622				
9	1.007467	-1.342859	0.127930			
10	-3.502435	-7.949626	-1.999576	-10.619951		
11	-0.240741	-0.924597	-0.889850	-1.887246	-3.753213	
12	-2.034872	-4.052033	-1.372241	-8.912682	-2.639692	-3.263457
13	-2.306003	-4.333632	-0.290565	-10.572677	-3.620535	-8.295492
14	-0.300113	-1.483337	-2.944124	-1.945225	-13.341797	-2.459579
15	-0.970331	-2.917477	-1.716808	-6.075354	-2.046407	-3.420218
16	-1.732537	-3.413270	-1.061172	-7.706998	-2.932204	-5.062631
17	-0.320514	-0.860419	-5.057534	-1.105343	0.087165	-1.368692
18	2.191991	-10.681564	-1.850065	-17.223844	-2.096990	-2.638148
19	-4.446236	-0.092506	-0.980315	0.574265	-0.746791	-1.066387
20	0.094167	-0.344945	-4.607331	-0.923433	-1.554106	0.582712
21	0.243195	0.177680	-4.557400	-0.527609	-1.750718	1.190394
22	-0.163031	2.347902	-0.593318	8.182926	-1.582904	-1.852959
23	-0.248795	1.772548	-0.085823	7.083871	-1.072684	-1.406417
24	-0.545507	-0.541362	-1.629891	-0.142179	-5.946854	-1.728058

	13	14	15	16	17	18
13	-2.823486					
14	-3.050821	-3.000899				
15	-4.183435	-3.510650	-5.192781			
16	-7.344532	-4.592162	-5.253183	-8.042821		
17	-0.157555	-2.009277	-3.196157	-1.796382	-8.481878	
18	-1.547881	-2.142747	-7.971597	-3.688425	0.197709	-2.106249
19	-1.823129	-1.812574	-1.497552	-2.140695	-0.504725	0.124549
20	-2.410839	-3.291188	1.541658	6.505778	-6.278585	-0.968351
21	-2.406913	-2.389717	-3.311176	-0.317815	-20.918886	0.517700
22	-1.174951	-1.328963	-3.140477	-1.736624	-0.329614	-35.966575
23	-0.721086	-0.886582	-1.568200	-0.988052	-0.116152	-23.320525
24	-1.026287	-7.364656	-0.139278	-0.714330	0.005293	-0.011022

	19	20	21	22	23	24
19	-10.128690					
20	-1.030345	-36.534988				
21	-0.739068	-105.881274	-40.105938			
22	-0.125856	-0.533756	-0.654786	-40.019684		
23	-0.212407	-0.286345	-0.595430	-158.494151	-47.195266	
24	-1.102454	0.383973	0.163435	-0.037493	-0.058357	-87.058017

CCSD(T)/aVDZ

	VIp	TS
1	68.697186	538.521771
2	210.797838	99.593917
3	255.894598	215.578844
4	441.189636	253.097146
5	458.407545	455.791964
6	560.747471	509.851270
7	634.134403	636.111986
8	833.699505	833.219063
9	912.142902	871.955717
10	944.050266	937.875746
11	1123.967707	1085.838143
12	1163.520557	1122.383342
13	1174.177565	1160.376604
14	1295.345948	1220.570313
15	1374.632081	1365.733146
16	1385.973002	1375.872322
17	1450.592466	1438.221066
18	1657.982025	1656.293023
19	1818.067295	1796.816799
20	3019.066146	3033.126934
21	3065.548646	3081.197075
22	3465.643579	3465.690454
23	3544.141590	3545.407237
24	3768.026944	3804.518521

VIp:

	1	2	3	4	5	6
1	3.204661					
2	49.439196	7.704130				
3	-0.161400	3.503294	0.647055			
4	2.391415	-0.248962	-0.667038	-1.702306		
5	-3.453639	2.513748	0.060441	-1.675517	-0.176745	
6	3.422458	-3.096021	-0.521420	-7.024977	1.706691	-1.284477
7	1.424737	0.569630	-0.012884	-0.062011	-0.374304	-1.521990
8	-0.251666	2.745715	-1.651921	-2.338193	-2.508576	-0.797451
9	-0.947463	6.422937	-0.104459	-0.142278	-0.529580	3.834223
10	0.116950	1.773373	2.268778	-1.181551	2.708582	-1.353433
11	0.693192	1.413873	-1.605545	-1.025090	-2.469873	-1.852025
12	-1.193248	0.895450	-4.044317	0.055260	-1.317962	0.016020
13	-1.948783	-1.230513	2.150678	-0.224793	0.008454	0.114266
14	-0.026224	0.287858	-0.417549	-12.277410	-1.520169	-1.339642
15	-0.379643	0.698166	-0.782529	-0.920540	-4.420546	-1.164851
16	-0.777972	-7.283707	-1.191396	-0.485458	0.368065	-1.116684
17	-0.837586	2.405462	-0.445668	-0.708665	-0.014463	-5.590493
18	1.069883	3.087036	0.193794	-0.097460	-0.090455	0.173704
19	-0.942181	-0.589491	-0.638945	-0.831985	-1.384731	-3.224530
20	0.162552	-1.623880	-0.047410	0.854820	0.441463	-1.658483
21	0.815209	-3.430389	0.030017	0.803944	0.630076	-1.301114
22	-9.781731	-3.657770	0.717857	0.215516	-0.082602	0.583525
23	-2.020580	-5.844232	0.830688	0.324279	-0.012368	0.656556
24	1.882652	0.040624	0.447971	-1.637830	0.034631	-2.657298

	7	8	9	10	11	12
7	-0.137865					
8	0.071005	-1.868645				
9	-0.092075	-1.526399	1.422114			
10	-2.924497	-4.594715	-2.507526	-10.378718		
11	-2.571822	-3.045508	-1.732047	-5.465736	-3.252783	
12	-1.444437	-3.382032	-0.760317	-16.202878	-8.403818	-3.175227
13	-0.194488	-1.913544	-0.247438	-2.938654	-2.854188	-4.931750
14	-1.113845	-3.354687	-3.078625	-1.549201	-8.444913	-4.488815
15	-2.877646	-5.583475	-0.170641	-7.010855	-5.484386	-4.742954
16	-0.302518	-2.207912	-2.409388	-5.727592	-2.750564	-4.199756
17	-0.300843	-0.789541	-9.898225	-1.217150	-1.478375	-0.700461
18	1.720535	-9.189962	-2.005705	-18.210387	-1.557806	-2.448905
19	-4.370207	-0.362613	-6.031845	1.218174	-4.009970	-1.984568
20	-0.067925	-0.295743	-4.864317	-1.001042	1.301151	-0.912446
21	0.128700	0.215495	-4.400529	-0.637339	1.060344	-0.847089
22	-0.251754	1.416032	-1.232913	7.928806	-1.092106	-1.781067
23	-0.336151	0.925828	-0.546578	6.183791	-0.721946	-1.354362
24	-1.026058	-0.065686	0.202935	-0.102350	-4.057315	-2.798513

	13	14	15	16	17	18
13	-1.248164					
14	-2.183951	-9.011133				
15	-7.682528	-8.408945	-7.793879			
16	-6.275576	-0.990236	-8.382786	-2.725263		
17	-1.431239	-0.435643	-1.925508	-5.045943	-1.811882	
18	-4.211435	-0.491785	-1.134557	-11.738792	0.066509	-2.230626
19	-1.267001	-4.536060	-2.067675	-0.502604	-0.579607	0.071764
20	-6.793442	0.414513	12.710088	-5.193140	-31.710620	-0.773515
21	-4.677687	-2.029392	-1.606313	-0.400755	-21.937950	1.450675
22	-3.310775	-0.694273	-0.726685	-4.492864	-0.529920	-36.340889
23	-2.524339	-0.188367	-0.598341	-2.167315	-0.322840	-24.715277
24	-0.258574	-7.615952	-0.603720	-0.064000	-0.023225	-0.033807

	19	20	21	22	23	24
19	-9.464589					
20	-0.876136	-30.982597				
21	-0.823680	-133.507232	-36.040763			
22	-0.248065	-0.639059	-0.648882	-40.989842		
23	-0.318798	-0.410191	-0.589162	-161.725612	-48.141446	
24	-3.894707	-0.288052	-0.246886	-0.005239	0.037365	-90.391492

TS:

	1	2	3	4	5	6
1	-13.655430					
2	-3.680888	3.328133				
3	-0.039311	42.605131	4.589623			
4	0.472970	1.387125	4.402238	0.653190		
5	1.065045	0.691866	2.758738	0.192847	-0.155355	
6	0.813836	-4.608550	-4.147098	-0.215769	-1.924309	0.633176
7	0.686415	4.625553	0.070128	-0.045934	-0.552567	4.032981
8	1.457582	-0.788331	2.718062	-1.323845	-2.862046	-1.933172
9	1.068156	0.189301	6.799250	-0.176177	-0.294648	-1.735611
10	0.994104	-0.576566	0.432715	2.461304	3.175484	-2.341456
11	-5.080266	0.162903	-0.143984	-1.008716	-0.309407	0.460136
12	2.115887	0.736473	-0.524781	-0.252348	-2.048146	-1.214815
13	2.524237	-0.424968	1.755523	-3.589429	-1.821315	-0.277345
14	-8.198715	-2.645135	0.615864	0.242338	-0.181066	-2.173626
15	0.773760	-0.794434	-0.331941	-1.526920	-4.687133	-1.308604
16	0.529808	-1.309136	-6.289291	0.399614	0.069537	-1.066939
17	0.954227	0.913250	2.103513	-0.650930	-0.156022	-0.328609
18	0.148361	1.307286	2.748530	0.164953	-0.086044	0.233483
19	0.148442	-0.714439	-0.722275	-0.481158	-1.115748	-2.219922
20	-0.718592	1.480330	-1.494619	0.113685	0.636901	-1.327518
21	-0.077506	0.039720	-2.461576	0.262291	0.646861	-0.857586
22	0.002503	-7.024618	-1.723327	0.581630	-0.158605	0.761203
23	-0.106503	-1.516836	-3.714889	0.715255	-0.093932	0.856496
24	14.652278	-0.001738	0.052668	-0.048736	-0.525078	-0.189009

	7	8	9	10	11	12
7	-0.125306					
8	-0.675727	-1.904615				
9	0.594383	-1.380147	0.049152			
10	-3.068914	-5.721876	-1.976035	-10.215880		
11	-0.738818	-1.230794	-1.229649	-2.321160	-3.743826	
12	-2.379055	-4.174988	-1.355012	-8.357568	-3.628760	-2.852849
13	-2.136628	-4.373584	-0.485176	-13.580448	-4.170497	-8.295547
14	-0.413526	-1.662754	-2.887453	-2.185431	-12.904433	-4.117399
15	-2.316323	-3.997905	-0.899724	-7.582942	-2.864582	-5.539035
16	-0.367421	-2.113017	-2.149180	-5.855509	-2.022544	-4.036127
17	-0.356673	-0.852075	-5.563438	-1.221669	-0.065891	-1.269510
18	1.655496	-10.672435	-1.913686	-19.542057	-1.829356	-2.450691
19	-4.496510	0.806575	-0.912619	1.784781	-0.849723	-1.320975
20	0.088238	-0.366117	-4.879183	-0.887051	-1.149888	-0.098306
21	0.229904	0.234452	-4.936342	-0.483099	-1.745266	0.879501
22	-0.225189	1.805049	-0.673615	8.258947	-1.436480	-1.700689
23	-0.312339	1.315821	-0.183817	6.956781	-0.974769	-1.307481
24	-0.676426	-0.751197	-1.735894	-0.234639	-5.717862	-3.069621

	13	14	15	16	17	18
13	-3.152685					
14	-3.026966	-2.906898				
15	-7.038227	-5.533658	-8.310056			
16	-4.063849	-2.828584	-8.516685	-2.962293		
17	-0.397716	-1.994683	-2.106538	-3.160968	-8.202496	
18	-2.165815	-2.212493	-1.785994	-9.746027	0.121703	-2.282768
19	-1.161024	-1.416186	-1.969666	-0.209880	-0.524823	-0.188479
20	-2.070426	-3.593578	11.412464	-5.702044	-8.197331	-1.091963
21	-1.779533	-3.054724	-3.089434	-2.511841	-21.673605	0.126755
22	-1.672611	-1.433388	-1.142050	-4.178589	-0.418636	-36.073072
23	-1.170839	-1.002694	-0.991943	-2.059129	-0.208804	-24.184110
24	-1.035746	-7.481549	-0.688550	-0.267517	-0.043099	-0.050251

	19	20	21	22	23	24
19	-10.792400					
20	-1.257824	-37.129576				
21	-1.256084	-110.090229	-40.806286			
22	0.054905	-0.620200	-0.778741	-41.174970		
23	-0.289586	-0.382202	-0.695330	-162.079833	-48.372762	
24	-1.198307	0.340467	0.121091	-0.076964	-0.076291	-88.802215

CCSD(T)-F12b/VDZ-F12

	VIp	TS
1	77.954826	554.853665
2	202.245796	107.867002
3	260.613404	211.331588
4	460.698926	257.618841
5	467.087520	465.184824
6	565.873034	517.460909
7	650.415550	653.782419
8	844.545175	846.257963
9	924.841678	881.922179
10	944.638125	939.300093
11	1145.875729	1095.981520
12	1177.654587	1141.010018
13	1192.824487	1177.605528
14	1307.108075	1236.767072
15	1398.294868	1390.866394
16	1406.535473	1396.491432
17	1476.642547	1464.072337
18	1684.361491	1683.269574
19	1855.852950	1834.660066
20	3035.596372	3050.970413
21	3078.276096	3091.347713
22	3511.694548	3512.800575
23	3585.580969	3587.931266
24	3816.048418	3851.931948

VIp:

	1	2	3	4	5	6
1	3.817243					
2	58.004717	10.328045				
3	-0.860588	3.415156	0.588145			
4	1.847052	-0.093902	-1.348049	-3.075854		
5	-4.078635	2.439869	0.091861	-2.118294	-0.302937	
6	5.414660	-1.720883	-0.438300	-6.449030	2.717434	-1.891024
7	1.180687	0.509313	-0.070985	-0.287178	-0.419506	-1.350748
8	-0.001585	3.572792	-1.731420	-2.512752	-1.040990	-0.248698
9	-0.828689	7.320520	-0.029034	-1.426270	-0.557195	0.395579
10	-0.039206	2.197946	3.003030	-0.813042	-0.061243	-1.317446
11	-0.000522	1.386207	1.115980	-0.385562	-1.918307	1.705812
12	-0.656161	1.494817	-4.444082	0.675311	-1.290826	0.272711
13	-1.278085	-1.287487	2.093420	0.351442	0.018768	0.437471
14	0.053792	0.122050	-0.488308	-10.313056	-2.629200	-1.984615
15	-0.478640	0.654578	-3.603812	-0.092861	-0.762956	-1.056801
16	0.061389	-8.114779	-1.092443	0.328145	0.499470	-1.462844
17	-0.887224	3.021942	-0.341510	-1.093966	0.052186	-1.534777
18	1.276324	3.148421	0.123017	-0.072629	-0.096155	0.229074
19	-1.003364	-0.708954	-0.617846	-0.815962	-1.574973	-3.230874
20	0.342885	-1.919707	-0.139227	0.741258	0.411173	-1.443223
21	0.900314	-3.687193	0.088091	0.904086	0.734545	-1.157700
22	-9.150531	-2.309670	0.652824	0.238506	-0.081721	0.632832
23	-2.212682	-6.212626	0.876578	0.336217	-0.026985	0.713388
24	1.631941	-0.004985	0.433007	-2.522562	-0.057532	-2.587943

	7	8	9	10	11	12
7	-0.509989					
8	-0.123620	-1.977291				
9	-0.014868	-2.125700	0.441292			
10	-3.283593	-4.556979	-2.786343	-10.843833		
11	-2.072098	-2.609539	-1.731564	-5.781712	-3.016519	
12	-1.473420	-3.237845	-0.456118	-13.709686	-9.185086	-2.690159
13	-0.205192	-2.171858	-0.071498	-3.537326	-2.663476	-5.018278
14	0.522782	-4.765666	-2.517941	-1.863185	-7.850116	-6.338837
15	-2.678661	-5.080220	0.321705	-3.587540	-7.474996	-4.756054
16	-0.303506	-2.939108	-2.462645	-5.280585	-3.075496	-3.649282
17	-0.279336	-0.828627	-5.730807	-1.057789	-1.548736	-0.596228
18	1.968364	-11.615443	-1.902912	-21.885676	-1.884519	-2.161684
19	-4.846583	-1.403436	-1.884899	0.454042	-3.647670	-2.771632
20	-0.216061	-0.589383	-4.275459	-1.257872	1.289221	-0.862984
21	0.089480	0.130841	-3.751789	-0.758462	1.364186	-0.894893
22	-0.225507	1.482199	-1.181142	7.976140	-1.328131	-1.503698
23	-0.359048	1.185826	-0.445792	6.442953	-0.959668	-0.984668
24	-0.976479	-0.332510	0.126548	-0.079340	-3.855106	-3.913896

	13	14	15	16	17	18
13	-1.311544					
14	-2.419295	-8.685180				
15	-8.758783	-7.550118	-9.251120			
16	-6.779376	-1.044801	-9.767491	-3.045392		
17	-1.992600	-0.494078	-2.428408	-6.522005	-1.990977	
18	-4.478860	-0.695984	0.872451	-6.675391	0.133676	-3.019137
19	-1.334944	-4.460680	-2.417071	-0.456358	-0.614923	0.050681
20	-6.629657	0.309866	13.852223	-4.551687	-32.094804	-2.891999
21	-4.213869	-1.792606	-1.561837	-5.156701	-21.446397	-4.253381
22	-3.084112	-0.429262	-0.719447	-4.123284	-0.644970	-36.703645
23	-2.224994	-0.052693	-0.573042	-1.752653	-0.452035	-22.938755
24	-0.314235	-5.519851	-0.590517	-0.115311	-0.077447	-0.045726

	19	20	21	22	23	24
19	-9.828437					
20	-0.989569	-29.678515				
21	-0.729786	-128.160926	-34.877933			
22	-0.349706	-0.894639	-0.623923	-39.388792		
23	-0.324544	-0.431369	-0.651590	-156.168573	-46.650847	
24	-3.512101	-0.599837	-0.353434	-0.181292	0.031791	-86.230381

TS:

	1	2	3	4	5	6
1	-14.906522					
2	-2.401255	1.860107				
3	-0.195630	28.342902	2.240081			
4	0.554188	0.871740	4.320657	0.567621		
5	0.980564	0.302708	2.600708	-0.001017	-0.442577	
6	1.536537	-3.674525	-3.120613	-0.159784	-1.568084	1.025185
7	0.500620	4.286927	-0.441703	-0.127908	-0.604767	3.699009
8	1.355019	-0.640522	2.857117	-1.245613	-2.553171	-1.879163
9	1.533092	0.578591	7.785226	-0.298380	-0.269653	-0.134774
10	0.928153	-1.060213	-1.992532	3.797271	0.067287	-2.449095
11	-4.896962	0.621638	0.907912	-0.544400	-0.033903	0.967177
12	2.454748	-0.415478	-0.075622	-0.169739	-1.874601	-0.836845
13	3.201954	-0.280711	1.079148	-4.456808	-2.073231	-0.434147
14	-6.989060	-1.300557	0.179634	0.390338	-0.224181	-1.950954
15	0.714532	-0.502671	-0.617670	-2.656503	-0.885302	-1.191100
16	0.728036	-0.876474	-6.340411	0.499077	0.252239	-1.528559
17	0.904332	1.287239	3.984016	-0.599132	-0.077125	-0.163983
18	0.130236	0.521901	2.724445	0.121216	-0.110541	0.318967
19	0.008302	-0.731566	-0.837635	-0.433213	-1.081137	-2.291272
20	-0.482276	1.296246	-1.532625	0.227487	0.653520	-1.338447
21	-0.137590	-0.280587	-2.906319	0.242314	0.787563	-1.090959
22	-0.029136	-4.389134	-0.122815	0.588326	-0.137345	0.805085
23	-0.122556	-0.844085	-2.095719	0.700542	-0.060509	0.857190
24	12.952003	-0.260121	-0.050650	0.018098	-0.440548	-0.252037

	7	8	9	10	11	12
7	-0.146290					
8	-0.795205	-2.038658				
9	1.127120	-1.459741	0.065358			
10	-3.406018	-16.860050	-2.117965	-10.136260		
11	-0.366590	-0.942877	-0.867461	-1.884777	-4.473989	
12	-2.010291	-4.115137	-1.179166	-9.160042	-2.759918	-3.224030
13	-2.216643	-4.512513	-0.432041	-11.588704	-3.682067	-8.424216
14	-0.390999	-1.703305	-2.855325	-2.330473	-13.116721	-2.740847
15	-2.210835	-3.658005	-1.058630	-3.423463	-3.368927	-6.260501
16	-0.314430	-2.590577	-2.720866	-5.318612	-2.245517	-4.057144
17	-0.320807	-0.888645	-5.092199	-1.005546	-0.103968	-1.401344
18	2.037073	-3.119193	-1.722814	-11.753455	-1.780441	-2.698389
19	-4.608945	-0.165358	-1.079240	0.502474	-0.958795	-0.971902
20	0.036572	-0.468477	-4.648097	-1.027570	-1.490381	0.476885
21	0.207980	0.074099	-4.485407	-0.526251	-1.205304	1.007790
22	-0.177237	2.461992	-0.726449	7.854920	-1.338146	-1.896686
23	-0.282230	1.929246	-0.204342	7.242851	-0.914763	-1.399280
24	-0.569106	-0.580665	-1.929264	-0.153099	-6.155343	-1.997140

	13	14	15	16	17	18
13	-3.077911					
14	-3.153334	-2.633855				
15	-6.493039	-6.933746	-9.622431			
16	-3.564507	-3.378768	-10.176874	-2.888093		
17	-0.478434	-1.147519	-2.145035	-4.116182	-2.248006	
18	-2.004316	-2.475080	-1.491743	-9.781558	0.123875	-3.041508
19	-1.556793	-1.916012	-1.849020	-0.211102	-0.561780	0.233987
20	-1.949425	-3.965780	14.052131	-5.604722	-31.965984	-0.970567
21	-2.001612	-2.770763	-3.147877	-3.622987	-22.550325	-0.787619
22	-1.381400	-1.461795	-0.941126	-4.080780	-0.546936	-37.000284
23	-0.855082	-0.979860	-0.806283	-1.858888	-0.347031	-22.507663
24	-1.421993	-6.751980	-0.734810	-0.181706	-0.014975	-0.042242

	19	20	21	22	23	24
19	-10.839179					
20	-1.279836	-30.371803				
21	-1.044638	-124.099847	-34.759819			
22	-0.321800	-0.647423	-0.654922	-39.462324		
23	-0.312154	-0.451249	-0.687784	-155.239870	-46.216800	
24	-1.373510	0.262622	0.214398	-0.072015	-0.090074	-84.957841

MP2/aVDZ

	VIp	TS
1	78.593470	548.575726
2	216.237948	102.796115
3	258.661203	216.734350
4	451.968298	254.131019
5	464.342127	461.496511
6	569.001359	514.474413
7	638.068590	639.240276
8	838.833072	836.177228
9	918.943871	873.680254
10	937.697316	930.260336
11	1127.335278	1075.259447
12	1174.014417	1126.957172
13	1181.276047	1169.760572
14	1285.550766	1216.119127
15	1377.005265	1368.809217
16	1391.804347	1380.399465
17	1457.158247	1441.945984
18	1657.650173	1655.474307
19	1812.079775	1790.133820
20	3063.138012	3077.043236
21	3117.960211	3131.302699
22	3509.118730	3509.949851
23	3597.899116	3600.116354
24	3779.902082	3815.949652

VIp:

	1	2	3	4	5	6
1	2.754412					
2	45.372847	6.575599				
3	0.175611	3.764262	0.622549			
4	1.891506	-0.693446	-0.573414	-2.731521		
5	-3.441176	2.817888	0.097902	-1.528938	-0.331960	
6	3.639748	-3.097367	-0.518807	-7.916226	1.989352	-0.809514
7	1.331020	0.723807	-0.000640	0.153931	-0.373257	-1.286019
8	0.069494	3.251619	-1.577733	-2.379562	-1.925468	-0.921390
9	-1.497123	6.336394	-0.107686	-2.495602	-0.755750	2.713910
10	0.245851	0.620910	-1.140669	-0.683957	3.755973	-1.394070
11	1.084020	1.461273	-1.563047	-0.376357	-2.824361	-5.350828
12	-1.490821	-0.269211	-0.812951	0.178723	-1.091088	0.112160
13	-1.827312	-1.065663	1.960243	-0.293800	-0.020160	0.020600
14	0.239042	0.442635	-0.530441	-9.951038	-1.983686	-1.664101
15	-0.274148	0.579745	-0.748324	0.543548	-5.385745	-1.003570
16	-0.941448	-6.063687	-1.019058	-0.928440	0.660315	-0.906104
17	-0.639750	2.445756	-0.416728	-0.665561	0.016787	-4.522523
18	0.967934	2.891504	0.210292	-0.088377	-0.024372	0.163948
19	-0.722003	-0.521866	-0.608292	-1.124431	-0.920092	-3.065483
20	0.720760	-1.415982	-0.036995	0.804422	0.380396	-1.402261
21	0.709186	-3.334414	0.052283	0.718057	0.571814	-1.231956
22	-8.233181	-2.383864	0.589757	0.274048	-0.131933	0.551350
23	-1.728917	-6.079868	0.668619	0.405723	-0.076448	0.605159
24	1.619758	-0.062536	0.366125	-1.447036	-0.160276	-2.791998

	7	8	9	10	11	12
7	-0.460670					
8	-0.163778	-1.970889				
9	-0.079981	-2.083161	0.572028			
10	-3.127752	-7.633133	-2.489607	-10.919272		
11	-2.959733	-3.095956	-1.659518	-3.276420	-4.024864	
12	-1.224494	-3.647726	-0.977610	-11.272418	-6.196928	-4.070091
13	-0.175961	-2.043901	-0.418390	-2.770786	-2.407675	-4.822263
14	0.326309	-3.414604	-2.126484	-1.855524	-10.920877	-3.618072
15	-2.626446	-4.843755	0.883544	-7.442966	-5.502874	-4.463782
16	-0.324451	-2.431686	-2.091624	-5.026148	-2.098866	-4.052915
17	-0.336520	-0.909208	-8.693045	-1.154756	-1.322181	-0.988682
18	1.907099	-10.756274	-1.908214	-17.988086	-1.038387	-2.861058
19	-4.629070	0.216543	-2.830694	1.444164	-4.923271	-1.085896
20	-0.090502	-0.562536	-4.419301	-1.107423	1.024359	-0.558867
21	0.108872	-0.051541	-4.169100	-0.760884	0.682861	-0.233056
22	-0.229153	1.762573	-1.228885	7.122881	-0.631390	-2.542097
23	-0.325310	1.172280	-0.581958	5.866383	-0.282435	-2.017909
24	-1.075892	-0.243253	0.152579	-0.081165	-5.815190	-1.995552

	13	14	15	16	17	18
13	-1.285371					
14	-1.782321	-8.637152				
15	-7.648637	-6.507671	-7.847004			
16	-6.412075	-0.982217	-8.476390	-2.725432		
17	-1.311269	-0.401001	-2.005047	-4.645209	-1.632835	
18	-4.557998	-0.325733	-5.443367	-9.764777	0.069950	-2.297657
19	-1.031473	-3.769076	-1.887000	-0.377499	-0.538734	-0.042820
20	-6.168336	-0.135602	15.534634	-5.097755	-31.184569	3.233852
21	-4.352070	-1.860691	-2.020160	-2.228298	-20.762468	-0.115543
22	-3.454509	-0.163230	-1.029705	-4.313384	-0.621111	-36.128355
23	-2.655274	-0.090698	-0.902591	-2.331074	-0.396336	-23.923944
24	-0.354362	-6.811560	-0.892942	-0.079536	-0.021602	-0.057440

	19	20	21	22	23	24
19	-8.504972					
20	-1.067632	-29.569277				
21	-1.120848	-126.192448	-34.295026			
22	-0.205845	-0.817581	-0.790104	-39.198322		
23	-0.396490	-0.588431	-0.746531	-153.185677	-45.936154	
24	-3.403015	-0.450823	-0.372355	-0.072741	-0.057068	-86.983843

TS:

	1	2	3	4	5	6
1	-13.703427					
2	-3.469340	2.451510				
3	-0.007927	29.041162	0.939705			
4	0.481919	1.233423	3.966820	0.653738		
5	1.076829	0.519456	2.984361	0.220390	-0.402795	
6	1.321265	0.408988	-4.501774	-0.209043	-1.739756	0.715640
7	0.814106	-0.065858	-0.007921	-0.078662	-0.609344	-0.631558
8	1.677460	-0.561581	2.573637	-1.057960	-2.381796	-1.922767
9	1.655552	0.657317	6.858664	0.203356	-0.247786	-1.753124
10	1.213750	-0.795996	-2.464867	-0.826963	5.311431	-2.409252
11	-4.118258	0.901457	0.951191	-0.676441	-0.266215	0.680986
12	3.049518	0.398484	-0.512236	-0.842177	-2.467409	-1.640470
13	1.742912	-1.149662	2.069129	-0.542055	-1.431516	0.286079
14	-5.878232	-1.404219	-1.060911	0.410338	-0.173231	-1.950803
15	0.818119	-0.575038	-0.223426	-1.554564	-5.748535	-1.134608
16	0.558381	-0.973674	-6.903004	0.207400	-0.292078	-0.977295
17	1.102756	1.383564	4.722969	-0.627333	-0.123282	0.029493
18	0.162551	0.833300	2.796764	0.213789	-0.007915	0.283098
19	0.084917	-0.684888	-0.778803	-0.460910	-0.828748	-1.957914
20	-0.611926	1.815796	-1.347048	0.146187	0.576261	-1.342537
21	-0.015023	-0.734804	-2.555419	0.235700	0.691889	-1.136654
22	-0.021369	-5.023894	-1.213029	0.483877	-0.164789	0.739786
23	-0.122699	-1.397065	-2.595307	0.553358	-0.090059	0.767955
24	12.667878	-0.196170	0.011979	-0.039730	-0.582113	-0.404566

	7	8	9	10	11	12
7	-0.118890					
8	-0.860412	-2.210106				
9	0.705842	-1.320410	-0.044596			
10	-3.312449	-9.767193	-1.974357	-10.331026		
11	-0.738503	-1.407100	-1.436291	-2.159164	-4.316123	
12	-3.047384	-4.753075	-1.698384	-5.785607	-4.341858	-3.089772
13	-1.609777	-4.269933	-0.550777	-9.573282	-3.940527	-7.429055
14	-0.435850	-1.857691	-2.878700	-2.223617	-11.353922	-3.714318
15	-2.167827	-3.608857	-0.927864	-8.041484	-2.782923	-5.265971
16	-0.413179	-2.591929	-1.889409	-5.061171	-1.965739	-3.492987
17	-0.389811	-0.975306	-5.003892	-1.292704	0.061139	-1.155354
18	1.872722	-12.674613	-1.809154	-19.149776	-1.578068	-1.934996
19	-4.359643	1.979291	-0.563423	2.253273	-0.880309	-1.571397
20	0.090318	-0.606382	-4.628744	-0.930821	-0.835417	-0.303232
21	0.243619	0.031615	-4.775955	-0.521754	-1.241383	0.563417
22	-0.160529	2.416099	-0.677399	7.198653	-1.263911	-1.334924
23	-0.229721	1.927453	-0.244765	6.738503	-0.864423	-0.880606
24	-0.772353	-0.819272	-2.437843	-0.307343	-7.625117	-4.007903

	13	14	15	16	17	18
13	-3.771615					
14	-2.912863	-2.176695				
15	-5.984943	-6.386272	-7.870847			
16	-6.132782	-3.304172	-8.100652	-3.093320		
17	-0.471015	0.429762	-2.131145	-2.864841	-7.460223	
18	-2.549984	-2.619588	-4.187152	-9.048539	0.143623	-2.313224
19	-0.568815	-1.100901	-1.643430	-0.240413	-0.475386	-0.062953
20	-1.649878	-3.982070	11.942896	-5.359046	-9.781848	1.475341
21	-1.462001	-3.418741	-3.851325	-3.208517	-21.476063	-0.355547
22	-2.366643	-1.829302	-1.449423	-4.121174	-0.489391	-36.237538
23	-1.764793	-1.348738	-1.234350	-2.331695	-0.254944	-23.648152
24	-0.739264	-6.594417	-0.771020	-0.295139	-0.036104	-0.047467

	19	20	21	22	23	24
19	-9.933017					
20	-1.469856	-32.885048				
21	-1.664457	-113.265619	-36.333276			
22	-0.193821	-0.831814	-0.825551	-39.357153		
23	-0.415616	-0.615015	-0.783062	-152.492626	-45.496073	
24	-1.139710	0.305391	0.206503	-0.076500	-0.083511	-85.617762

Frequencies and Anharmonicities for Tri-Deuterated Glycine

B3LYPD3/aVTZ

	VIp	TS
1	71.780359	414.113275
2	158.844537	86.777464
3	246.167086	163.689480
4	356.829231	242.495567
5	432.361257	441.387066
6	545.959324	495.073098
7	612.591466	613.982657
8	733.568895	722.518901
9	803.283431	738.730593
10	827.459216	858.094551
11	942.439039	867.652823
12	1080.698780	1072.073085
13	1089.097701	1097.310732
14	1203.662044	1131.290903
15	1239.667594	1237.493741
16	1321.692806	1308.811399
17	1383.014952	1380.644652
18	1465.982084	1448.588615
19	1837.490978	1831.985510
20	2531.652192	2531.780921
21	2629.497984	2630.665696
22	2748.663112	2776.336996
23	3012.218548	3024.767133
24	3044.411559	3058.967724

VIp:

	1	2	3	4	5	6
1	5.652123					
2	19.328787	-0.595786				
3	4.554571	6.837486	0.597382			
4	-0.305303	-0.002306	-0.508735	-4.156995		
5	-2.892296	1.210953	-0.102096	-1.333172	-0.247500	
6	3.163752	-1.670771	-2.456147	-3.417670	1.002755	1.293684
7	0.468609	0.759580	0.123683	0.010276	-0.505263	-1.324308
8	1.041270	-0.443263	-1.860607	1.417746	-1.145994	-1.303351
9	0.863454	2.868122	2.113584	-0.674059	-0.510530	1.915948
10	-0.289923	0.159093	-2.476911	1.497454	-3.382624	-1.335990
11	-1.449472	-0.595849	-1.291844	-0.885765	-3.877351	-0.138012
12	-1.330335	-3.838509	-0.402505	-1.191558	-0.388404	-2.423804
13	-1.489644	-0.961505	-0.650119	-0.736657	-0.764056	-2.346404
14	1.549904	1.216001	-1.583271	-4.938014	-3.675012	-2.203960
15	0.410027	-0.195016	0.561108	-0.171346	-0.192938	-0.232334
16	-0.995477	-1.928070	0.920735	-0.044267	-0.467219	-0.015889
17	-1.999097	0.563388	-0.489151	-0.289670	0.870855	-0.268653
18	-0.806085	-0.322978	-0.127824	-0.795848	-0.317846	-2.147554
19	-0.862074	-0.718361	-0.522205	0.131066	-0.063036	-2.623340
20	-4.437595	-0.289492	0.454124	0.028391	-0.090952	0.575934
21	-2.363165	-0.017125	0.600737	0.087653	-0.028170	0.703507
22	0.832328	0.051454	0.190279	-1.525967	-0.439546	-0.222525
23	0.784446	-1.111696	-0.040417	0.868361	0.372236	-1.524600
24	0.128734	-2.639924	0.111156	0.675403	0.607079	-1.070857

	7	8	9	10	11	12
7	-0.434151					
8	-5.802982	-5.952796				
9	-0.448780	-4.159581	-0.278239			
10	-3.870828	-9.086642	-6.069586	-3.513483		
11	-1.116601	-2.862466	-2.524194	-6.565218	-2.209154	
12	-0.846821	-2.393436	-2.089094	-1.791599	-0.824913	-1.342369
13	-0.015882	-1.052891	-2.113370	-0.822450	-1.360690	-2.051568
14	-3.166963	-3.584597	-0.512302	-6.482315	-8.584628	-1.089854
15	-4.323281	-8.009900	-4.407456	-0.346829	-0.491700	-3.317282
16	-0.383010	-0.437138	-1.609834	-1.944815	-0.425657	-0.959331
17	-0.462191	-0.668555	-0.878019	-4.396032	0.361038	-2.970186
18	-0.215915	-1.201213	-2.552147	-0.268256	-0.450940	-7.037523
19	-3.284610	-0.417518	-0.956815	1.425789	-4.745189	-1.970631
20	1.134115	5.066345	-2.046852	1.345986	0.166697	-1.125794
21	0.766119	4.863333	-0.607560	1.810116	0.313437	-0.042686
22	-0.497481	-0.315684	0.059666	-0.418663	-5.847509	-0.067839
23	-0.472739	-0.854267	-1.356905	0.143349	0.658721	-5.343753
24	-0.244339	-0.589892	-1.136790	0.746660	0.806231	-3.541721

	13	14	15	16	17	18
13	-4.546064					
14	-3.033238	-6.402032				
15	-5.558029	-1.134489	-1.404852			
16	-3.863876	-2.875163	-5.716640	-3.802367		
17	-3.268985	-5.445856	-0.820502	-17.054586	-10.845710	
18	-2.581598	0.087175	-0.373261	-7.130662	-2.391451	-1.717894
19	-0.008208	-2.663192	-0.098907	-1.066128	-0.728078	-0.734263
20	-3.799242	0.049604	-19.300275	-1.484883	-1.867869	-0.730499
21	-3.468274	0.340951	-6.900633	1.413568	-0.368131	-0.352364
22	0.161255	3.315996	0.058473	-0.211261	-0.474527	-0.004649
23	1.128988	-0.256514	-0.553362	-6.823880	18.970251	-31.115198
24	1.950473	-1.006333	-0.733529	-2.879225	-2.503946	-19.808910

	19	20	21	22	23	24
19	-10.092004					
20	-0.080305	-20.242800				
21	-0.285795	-80.339471	-26.444330			
22	-2.159008	0.004164	0.043830	-44.577918		
23	-1.202415	-0.527872	-0.354120	-0.263300	-29.145131	
24	-1.349927	-0.509167	-0.540082	-0.217780	-127.936534	-34.290073

TS:

	1	2	3	4	5	6
1	-8.100481					
2	-1.712429	7.508046				
3	-0.307111	15.128598	-1.528031			
4	0.468684	2.009410	3.095186	0.651941		
5	1.122963	0.817457	1.503426	0.284733	0.537045	
6	1.315076	-2.091867	-1.863528	0.256543	-1.217134	0.663240
7	0.522008	1.307242	0.790378	-1.626047	-0.705918	1.712336
8	1.070578	0.746563	1.119088	-1.414781	-0.962451	-0.720933
9	-0.954894	0.411213	1.766233	-0.730332	-0.263856	-1.347991
10	-4.796777	0.226175	-2.546686	0.390269	-0.204062	-1.461539
11	1.015117	0.132147	-2.443059	0.738187	-7.947297	-2.832622
12	1.600961	-0.453871	-0.600355	-2.612968	-1.436322	-0.517145
13	-1.135311	-0.663618	-2.134678	-0.240016	-0.607927	-3.505849
14	3.728784	-0.024641	-0.683210	-1.377529	-3.228871	-2.252729
15	0.151383	0.690718	-0.319912	0.558849	-0.005922	0.032921
16	0.641153	-1.642333	0.281159	0.701908	0.781020	-0.551967
17	0.424986	-0.913566	0.182035	0.025759	-1.246513	0.811670
18	0.926132	1.373163	0.463544	-0.625741	-0.081085	-1.843974
19	-0.990381	-0.728509	-0.898122	-0.430958	1.163467	-1.200644
20	-0.003787	-3.391142	-0.308282	0.364320	-0.110558	0.524083
21	-0.077590	-2.156072	0.634807	0.445019	-0.039949	0.595634
22	6.631777	-0.126972	-0.056221	-0.001947	-0.433387	-0.688562
23	-0.503263	1.025422	-1.018392	0.142145	1.003053	-1.248408
24	0.220343	-1.315107	-0.809329	0.350286	0.566056	-0.093399

	7	8	9	10	11	12
7	-0.692546					
8	-4.798277	-4.785677				
9	-2.952084	-4.166673	-1.847224			
10	-0.439673	-3.572436	-5.018419	-2.570461		
11	-2.506412	-7.528232	-4.224698	-5.494594	-3.298397	
12	-0.585383	-1.241531	-1.655571	-1.109065	-3.323529	-3.751191
13	-1.848740	-2.469370	-3.527819	-3.334718	-0.915226	-2.712695
14	-2.680155	-2.803545	-1.636306	-1.427980	-5.567934	-6.646032
15	-4.850827	-7.021902	-3.448625	-2.928871	-0.368168	-4.626120
16	-0.264218	-0.262122	-0.536824	-1.296144	-1.436998	-3.406377
17	-0.422634	-0.492497	-0.156725	-0.939889	-2.935352	-3.433316
18	-0.415783	-1.988485	-5.131542	-0.085311	0.056363	-2.287124
19	-3.173241	-0.128067	-0.618884	-0.185946	-0.124701	-0.496732
20	1.434089	3.942140	0.926208	-1.453411	1.194113	-3.017788
21	1.107984	4.155906	1.496373	-0.490953	1.696378	-2.686019
22	-0.276706	-1.928921	-1.057472	-2.472227	-0.349885	-0.283012
23	-0.531593	-0.863919	-2.204007	0.413025	0.425891	0.958876
24	0.114991	-1.303505	-1.342419	0.198141	0.954762	1.896191

	13	14	15	16	17	18
13	-1.316473					
14	-2.540209	-4.170514				
15	-3.273521	-1.399533	-1.384109			
16	-1.417790	-1.216583	-4.861159	-3.300973		
17	-2.206447	-4.975420	-0.895989	-15.981634	-10.723405	
18	-7.222186	-0.593845	-0.168951	-3.997500	-1.727951	-2.732049
19	-2.292778	-3.146264	-0.062281	-0.314574	0.248628	-0.736304
20	-1.051507	-0.536385	-19.792906	-1.595177	-2.345843	-0.615580
21	-0.053421	-0.134231	-7.619564	0.598069	-0.558887	-0.294288
22	-0.145880	-1.618155	-0.034529	-0.121092	-0.282711	0.017353
23	-4.053597	-0.685021	-0.748374	-7.672452	16.364792	-30.351372
24	-3.082000	-1.024198	-1.195305	-5.508163	-1.393545	-19.404353

	19	20	21	22	23	24
19	-10.758094					
20	-0.067697	-20.313666				
21	-0.284527	-80.515087	-26.568860			
22	-1.045595	-0.026265	-0.041621	-44.192311		
23	-1.563334	-0.496464	-0.357973	0.328708	-39.802828	
24	-1.827692	-0.582915	-0.596356	0.179222	-87.268322	-42.619561

CCSD(T)/aVDZ

	VIp	TS
1	60.572033	397.731064
2	167.003440	87.990207
3	238.842149	174.251470
4	338.072187	236.902609
5	425.734870	434.595706
6	538.641785	486.653886
7	605.091599	606.564881
8	749.029374	731.797936
9	789.071126	746.706160
10	853.736349	866.123171
11	943.092651	876.722679
12	1071.962665	1072.819391
13	1088.241658	1091.974064
14	1209.783547	1143.057933
15	1225.118464	1222.217462
16	1303.365329	1291.988234
17	1380.536831	1375.682708
18	1450.247440	1438.051977
19	1810.572085	1796.328990
20	2506.458555	2506.513518
21	2607.980703	2609.072484
22	2742.168538	2768.727956
23	3019.101002	3033.238767
24	3065.898011	3081.662301

VIp:

	1	2	3	4	5	6
1	4.471918					
2	14.387100	-1.959600				
3	5.060889	6.505032	0.547918			
4	1.569845	0.479123	-0.251908	-1.292833		
5	-2.429999	1.518999	0.366549	-0.992345	-0.129451	
6	3.596871	-1.688705	-2.238985	0.773730	0.889806	0.593694
7	0.450929	0.217519	-0.445332	0.215436	-0.123732	-1.358984
8	0.187169	-0.841185	-1.716769	-0.197517	-1.029940	-1.373091
9	-0.804514	6.036158	1.990905	-0.080233	-0.055389	2.453846
10	1.639745	-0.940044	-1.323128	-3.900766	-2.521926	-5.025075
11	-1.046649	-0.825258	-1.101525	-2.051258	-2.446322	-1.140291
12	-0.910160	-2.134029	-0.345128	-0.789815	-0.266545	-2.390404
13	-1.609279	-1.196439	-0.521443	-0.705542	-0.659779	0.339383
14	1.919385	0.883654	-0.153145	-4.059823	-2.392630	-1.453027
15	0.370173	-0.812104	0.380614	-0.644416	-0.540331	-0.498169
16	-2.597728	-1.034153	1.030669	0.241658	0.405513	0.313244
17	1.078961	0.443287	-0.897295	-0.444541	-1.459113	-0.621543
18	-0.496582	0.328548	-0.595620	-0.427192	0.022783	-2.294023
19	-0.923395	-0.519406	-0.538668	-0.244363	0.830353	-3.078291
20	-5.467215	-1.140281	0.526459	0.007392	-0.094477	0.497100
21	-2.030639	-0.429001	0.592384	0.092343	-0.074582	0.554678
22	1.071990	0.078909	0.251750	-1.223976	-0.196654	-0.290026
23	0.933345	-0.913146	-0.099871	0.836596	0.340954	-1.777764
24	0.286208	-2.231820	0.006575	0.660148	0.522562	-1.365230

	7	8	9	10	11	12
7	-1.021521					
8	-4.446071	-5.610029				
9	0.984306	-3.342530	0.483921			
10	-1.923604	-10.452187	-3.240823	-3.577833		
11	-1.039894	-3.006769	-2.453735	-8.411890	-2.336045	
12	-0.488620	-2.210935	-2.094233	-1.131569	-0.614224	-1.344486
13	-0.276520	-0.506398	-1.894283	-0.864066	-1.400805	-2.034919
14	-1.150770	-3.644853	-0.378540	-5.496906	-6.417290	-1.086720
15	0.993896	-5.928890	-3.487396	-0.302412	-1.374463	-3.864992
16	-0.319677	-0.424624	-0.900413	-1.250252	0.201114	-0.750672
17	0.387862	-0.240895	-1.171687	-4.210378	-1.121276	-2.886070
18	-0.242719	-0.971306	-2.028102	-0.365279	-0.285902	-7.609329
19	-6.195719	-0.582158	-0.967255	5.265042	-1.291603	-2.014282
20	0.510367	4.960620	-2.491141	1.531686	0.210537	-1.563466
21	0.097024	3.833115	-1.122250	1.759198	0.324230	-0.578004
22	-0.524412	-0.227755	0.086042	-0.431750	-6.926354	-0.083318
23	-0.253497	-0.626925	-1.778064	-0.174868	0.582378	-6.153362
24	-0.062147	-0.401478	-1.643929	0.245288	0.691356	-4.614703

	13	14	15	16	17	18
13	-4.330688					
14	-3.443216	-4.542289				
15	-5.533060	-5.428951	-1.323514			
16	-3.445800	-3.188368	-4.910560	-2.948509		
17	-4.266747	-7.692892	-1.447774	-11.079334	-9.162071	
18	-1.956733	-0.189854	-0.266285	-4.409765	-2.065424	-1.775226
19	-0.453530	-3.323759	-0.469814	-0.874653	-0.034937	-0.573236
20	-4.502240	0.656510	-16.184800	-1.861476	-1.993238	-0.500395
21	-4.478788	-0.346881	-8.482973	0.491939	-0.612001	-0.150882
22	0.006994	1.575351	0.302034	-0.035427	-0.262228	-0.007260
23	0.526203	-2.412527	-0.256899	-8.824998	15.528353	-31.707925
24	1.207899	-3.325355	-0.219148	-5.831916	-1.966465	-22.243667

	19	20	21	22	23	24
19	-9.889065					
20	-0.056693	-21.409664				
21	-0.225704	-84.498982	-27.491784			
22	-2.543360	0.011500	0.022976	-48.020983		
23	-2.030724	-0.514284	-0.335158	-0.261972	-30.993723	
24	-1.055441	-0.449670	-0.417836	-0.204175	-133.962679	-36.043404

TS:

	1	2	3	4	5	6
1	-7.639067					
2	-1.814413	5.707074				
3	-0.368015	11.029801	-2.351672			
4	0.347663	1.558238	2.509862	0.539026		
5	0.977938	0.763666	1.452908	0.414599	-1.103378	
6	1.206057	-1.709715	-1.925683	-1.065083	-1.258292	0.781130
7	0.581946	1.240618	1.167976	-0.217828	-0.361421	1.020983
8	0.743692	0.675454	2.452956	0.239715	-0.548018	0.826958
9	0.271609	0.265352	0.646823	-0.918706	-0.692801	-0.725046
10	-4.665312	-0.142130	-1.723755	0.274106	0.168821	-1.541348
11	0.600528	0.309907	-3.013483	-0.643399	-0.225359	-3.721265
12	1.159302	-0.414763	-0.219896	-2.170905	-1.199387	-0.248487
13	-1.705637	-0.232431	-1.658856	-0.046975	-0.427676	-2.358344
14	3.709571	-0.308916	2.095916	-1.364685	-3.013153	-1.984063
15	0.150762	0.726128	-0.278759	0.720919	0.054069	0.001180
16	0.346380	-0.017678	-2.170237	0.642165	0.163732	-0.753820
17	0.477641	-2.070171	0.077667	-0.425825	1.183938	0.918716
18	0.686493	1.120672	0.656843	-0.803791	-0.259062	-1.973533
19	0.065969	-0.635783	-0.591009	-0.453907	-1.782361	-1.684200
20	0.012791	-2.749123	-0.210149	0.447289	-0.130683	0.501769
21	-0.059621	-1.718193	0.373104	0.503595	-0.072064	0.516256
22	6.972451	0.003650	-0.017821	-0.023607	-0.403205	-0.604323
23	-0.467406	1.757100	-0.508524	0.120628	0.760070	-1.069473
24	-0.042832	-0.902613	-0.834517	0.191110	0.549852	-0.503145

	7	8	9	10	11	12
7	-0.181956					
8	-1.550252	-1.506181				
9	-3.589653	-3.682037	-3.453407			
10	0.126891	-3.658889	-2.158485	-2.943874		
11	-2.696850	-5.513559	-7.915978	-4.216391	-3.448144	
12	-0.721797	-0.636532	-1.313011	-0.691495	-2.633714	-3.935685
13	-0.464609	-2.803070	-2.525789	-3.257805	-0.546240	-2.151733
14	-3.185248	-1.537031	-2.283332	-1.751544	-5.341662	-5.303755
15	-2.847347	-3.616505	-4.436431	-1.961922	-0.180660	-5.345112
16	-0.306098	-0.168162	-0.460148	-1.435564	-0.967280	-3.184295
17	0.166689	-0.351000	0.047397	-0.684364	-2.644391	-4.635536
18	-0.421111	-4.631687	-2.767866	-0.027065	-0.330575	-1.653974
19	-4.033873	-0.433554	-0.429682	0.446340	0.737062	-0.790118
20	0.665166	1.537805	2.685541	-1.299091	1.405324	-3.974340
21	0.303819	1.732365	2.575710	-0.558356	1.746966	-3.724610
22	-0.454462	-2.454037	-0.246779	-4.407587	-0.227501	-0.324063
23	-0.255576	-1.478359	-1.626660	0.682572	-0.660933	0.676217
24	0.179118	-2.056612	-1.069214	-0.310310	0.885086	1.262125

	13	14	15	16	17	18
13	-1.208473					
14	-2.626340	-4.095462				
15	-2.730410	-1.311811	-1.339658			
16	-1.072081	-3.876595	-4.664361	-2.740565		
17	-2.062148	-5.704038	-1.234681	-13.970991	-9.124268	
18	-7.683241	-0.281115	-0.113685	-2.459244	-1.868813	-8.198003
19	-2.352195	-2.933779	-0.141366	1.368309	-2.698695	-0.556320
20	-1.302434	-0.738584	-19.377321	-2.142104	-2.747838	-0.514011
21	-0.374857	-0.386497	-9.207598	-0.174726	-0.883442	-0.082357
22	-0.753929	-1.502301	-0.061995	-0.145583	-0.408566	-0.034028
23	-4.968704	-1.316751	-0.875725	-9.445992	13.763874	-8.305822
24	-3.825730	-2.372964	-1.460185	-8.838738	-1.854933	-21.896465

	19	20	21	22	23	24
19	-10.848379					
20	-0.039816	-21.497614				
21	-0.215501	-84.707173	-27.630454			
22	-0.840543	-0.047748	-0.036965	-47.158556		
23	-1.249744	-0.524215	-0.367249	0.223732	-37.011726	
24	-2.886896	-0.509883	-0.466914	0.097517	-110.648976	-40.704097

CCSD(T)-F12b/VDZ-F12

	VIp	TS
1	69.610000	408.130395
2	159.900000	91.319190
3	242.930000	169.369112
4	352.510000	241.596055
5	433.260000	443.022351
6	543.920000	494.171858
7	617.100000	619.928762
8	754.300000	738.220471
9	799.520000	752.449475
10	855.510000	874.360007
11	958.320000	888.995732
12	1087.740000	1090.899852
13	1104.490000	1109.003789
14	1232.150000	1164.448934
15	1247.590000	1243.717800
16	1324.630000	1315.691253
17	1403.280000	1399.241084
18	1476.430000	1463.784814
19	1848.190000	1834.234549
20	2539.840000	2539.492236
21	2640.360000	2641.254173
22	2778.030000	2803.115060
23	3035.630000	3051.284583
24	3078.260000	3091.801771

VIp:

	1	2	3	4	5	6
1	0.787148					
2	-3.820874	-18.538741				
3	2.723570	-16.612760	0.259424			
4	2.538683	2.910487	-0.840949	-20.646645		
5	1.403054	2.438445	-0.795274	-7.044257	-0.649547	
6	1.125060	-1.970460	-0.587719	-0.118014	0.683208	0.534057
7	-2.378056	-1.068791	-0.197789	-2.034044	-0.362784	-1.644517
8	-7.924410	-14.831003	-5.988691	8.895949	-1.670846	-1.914658
9	-2.558235	-2.908030	-1.242580	-0.015127	-0.156443	0.757611
10	0.087998	-4.862918	-3.481143	8.340448	-4.196236	-2.125154
11	-1.815806	-0.007610	-2.576080	-1.045368	-5.328336	-1.840279
12	-0.271724	-4.483060	-0.646135	-0.427912	-0.346383	-2.257007
13	0.831975	2.765492	-0.067607	-2.489968	-1.112403	0.495349
14	0.913246	-0.442976	0.622363	-3.052155	-1.890140	-1.540480
15	0.472170	0.091999	0.060736	-0.826204	-0.759708	-0.694293
16	1.397142	0.527250	1.387077	0.371365	0.479160	0.350781
17	2.637633	2.296190	-0.480590	-0.274559	0.970156	-0.298863
18	-0.397083	1.658861	-0.641811	-1.055056	0.086212	-1.862114
19	-2.102765	-1.273876	-1.943358	-1.778856	0.107701	-2.977026
20	-0.275867	9.706361	2.972221	-0.976688	-0.029726	1.199927
21	9.428161	25.066489	3.683191	-1.169369	0.035899	1.285347
22	-2.639014	-3.601537	-0.591389	17.180958	3.145776	-0.909480
23	0.368553	-1.713275	0.204564	1.039044	0.090596	-1.011590
24	-1.331507	-2.583735	0.441406	1.015639	0.345158	-0.098433

	7	8	9	10	11	12
7	-2.189553					
8	-13.851774	-10.495474				
9	-1.120174	-11.017576	0.155664			
10	-5.404564	-13.662041	-5.441026	-4.285613		
11	-1.301748	-4.389603	-1.339016	-12.295535	-3.685523	
12	-0.986426	-2.969514	-2.436821	-1.177205	-0.569862	-1.303756
13	0.233904	1.070374	-1.346848	-1.810281	-1.789096	-2.033983
14	-1.905068	-3.336668	-0.794230	-5.576272	-5.544917	-1.887619
15	3.102899	-4.520782	-4.021682	-0.867393	-1.941373	-3.460517
16	-0.247250	0.170637	-0.966162	-1.504674	0.754108	-0.551954
17	0.229056	-0.405611	-1.166350	-4.030647	0.702581	-2.759781
18	-0.296153	-1.052883	-2.016024	-0.488348	-0.289286	-7.422517
19	-4.671464	-1.915951	-1.142326	1.583325	-4.705604	-1.907649
20	3.735661	12.237729	0.519712	2.233735	0.055575	-0.635090
21	4.145901	13.753628	1.389118	3.167358	0.494325	0.367502
22	-1.801151	-1.751157	-0.190775	3.874187	-0.065136	-0.282782
23	-0.439141	-1.007568	-1.722501	-0.582137	0.367802	-5.847588
24	-0.166007	-0.529230	-1.782076	-0.009504	0.781566	-3.984724

	13	14	15	16	17	18
13	-4.656252					
14	-3.293516	-3.919658				
15	-5.377169	-7.746961	-1.400805			
16	-4.008050	-3.587517	-4.929894	-3.200824		
17	-3.850725	-7.246965	-1.683280	-12.572632	-10.823236	
18	-1.895192	-0.272817	-0.367576	-5.166008	-2.410147	-1.794435
19	-0.189666	-3.427311	-0.626880	-0.420205	-0.063411	-0.610696
20	-6.052472	2.360506	-13.913988	-2.740138	-2.416247	-0.421314
21	-6.106896	-0.330636	-7.243587	0.185017	-0.870290	-0.069783
22	2.166860	0.761693	0.335463	1.212304	-0.601300	0.239688
23	-0.118659	-1.132381	-0.160830	-8.789046	18.759459	-32.043763
24	0.683322	-3.096418	0.047080	-5.420305	-1.510314	-22.280413

	19	20	21	22	23	24
19	-10.708125					
20	0.260162	-24.160735				
21	0.505645	-94.096683	-31.859235			
22	-0.682168	1.457178	2.033858	-54.393554		
23	-0.984296	-0.598142	-0.148303	-0.682523	-29.901588	
24	-0.917196	-0.228290	-0.235130	-0.398389	-129.219093	-35.221322

TS, with finite difference step size 0.5:

	1	2	3	4	5	6
1	-6.659062					
2	8.028401	-73.417290				
3	4.374557	-70.804320	-20.488953			
4	1.302787	0.620271	-7.572440	0.243388		
5	0.537303	-3.340976	0.196517	-0.192519	-0.294136	
6	1.823520	-3.759195	-2.813152	-0.743042	-0.709865	0.453792
7	1.485291	-3.006921	-1.482053	-2.028199	-0.587406	1.560120
8	0.931219	-24.941739	-4.974861	-2.593427	-0.897589	-0.245784
9	1.361671	0.754169	-5.975120	-2.991574	-1.240652	-0.861662
10	-8.565223	3.752616	-4.613336	0.099092	0.101822	-4.125534
11	2.178647	-10.314241	-11.609642	-0.783266	-4.651229	-4.460844
12	0.259920	2.326967	2.390291	-1.560173	-0.869379	0.361380
13	-1.874654	-2.103572	-3.646662	-0.616186	-0.591328	-3.410380
14	5.596268	-3.541020	-0.340394	0.733818	-3.132768	-2.058650
15	0.677972	-15.251449	-4.343237	0.199480	-0.377389	-0.058590
16	1.029615	-1.432862	0.776096	0.991382	0.294715	-0.977772
17	1.007161	-2.114540	0.645373	-2.594492	2.582409	1.988063
18	2.078910	-10.312652	-5.416073	-1.068295	-0.435010	-2.027315
19	0.136660	-6.295685	-2.428609	-1.548536	-2.892973	-1.705560
20	-2.703867	37.021992	17.344628	3.155237	0.912814	1.019674
21	-2.908031	41.815075	27.828228	3.633331	1.022091	1.078765
22	12.309155	-12.410952	-4.897117	-0.436487	-0.404943	-0.153784
23	-3.985584	25.582968	8.836721	0.886115	1.146881	0.934565
24	-2.906789	14.270427	5.280524	0.751821	1.192598	1.158188

	7	8	9	10	11	12
7	-1.028443					
8	-4.523175	-3.015309				
9	-10.548682	-6.804661	-7.924862			
10	-0.451755	-4.661621	-7.106495	-4.068037		
11	-3.594774	-10.152036	-8.922546	-5.247195	-3.135945	
12	0.168280	-0.541395	0.400422	-0.779380	-2.278779	-3.993425
13	-2.033164	-3.345991	-3.655434	-4.335747	-0.253109	-1.983899
14	-2.993042	-1.888253	-2.832045	-1.275649	-5.609101	-4.256941
15	-2.718284	-5.239771	-2.818725	-2.202291	-1.011447	-5.355732
16	-0.188344	0.036588	-0.538090	-1.139083	-1.003566	-3.027923
17	0.362382	-0.405583	0.264584	-0.152206	-1.540399	-3.909321
18	-0.601236	-7.705555	-3.483266	0.383321	-0.786726	-1.069660
19	-4.163849	-0.967831	-1.010149	0.190271	-0.930926	0.210123
20	4.101662	8.194205	7.581198	-1.131774	4.148731	-5.087663
21	4.366052	8.817505	8.777595	-0.533943	4.827413	-5.071110
22	-0.906163	-2.347525	-0.458882	0.791128	-1.499234	0.247018
23	-0.006994	0.145729	0.391613	-0.893970	0.900383	-0.703896
24	0.148361	-0.626479	0.475716	-0.175793	0.971834	0.318314

	13	14	15	16	17	18
13	-1.363153					
14	-2.252057	-4.326643				
15	-3.050153	-1.478556	-2.373114			
16	-0.962917	-3.545043	-5.585632	-3.281693		
17	-1.979009	-8.554174	-1.789584	-14.821528	-10.577921	
18	-7.756191	-0.534093	-1.044127	-6.531733	-2.614322	-2.829939
19	-2.572213	-4.204782	-0.381283	0.366060	-3.308473	-1.101856
20	-0.413655	0.563646	-16.517711	-1.999567	-2.325205	2.060035
21	0.527871	0.937063	-5.827116	0.204092	-0.621922	2.396188
22	-0.445278	-2.623965	-1.035569	-0.742432	-0.869453	-1.527628
23	-3.951965	-0.231382	1.023785	-8.285137	17.761226	-28.711153
24	-2.670480	-1.417311	-0.102003	-5.930450	-1.648725	-20.678750

	19	20	21	22	23	24
19	-11.097706					
20	1.243803	-26.958762				
21	1.244364	-99.751009	-34.369726			
22	-0.995696	2.917720	2.939584	-48.577449		
23	-0.290142	-5.443039	-5.194760	3.829574	-34.551703	
24	-0.834759	-3.766337	-3.737815	2.781596	-131.277072	-37.858193

TS, with finite difference step size 0.7:

	1	2	3	4	5	6
1	-5.101907					
2	7.500136	-65.671397				
3	3.786544	-45.457459	-17.489199			
4	1.278350	-2.827947	-10.357818	0.239722		
5	0.528330	-3.293748	0.244317	-0.205701	-0.295917	
6	0.623788	-3.882885	-2.965394	-0.741187	-0.732966	0.197786
7	1.430657	-2.865930	-1.315018	-2.049707	-0.596070	1.560431
8	0.056204	-23.381744	-4.449907	-2.570483	-0.923847	-0.736653
9	0.686204	0.176994	-5.602534	-2.973007	-1.245444	-1.113849
10	-10.050648	3.163437	-4.567523	0.044325	0.042435	-4.499853
11	2.090553	-9.610322	-10.732122	-0.805529	-4.654807	-4.449423
12	0.224233	2.312173	2.108463	-1.577323	-0.873941	0.342786
13	-2.041344	-2.258115	-3.803051	-0.631486	-0.611435	-3.712438
14	5.439513	-3.560968	-0.393807	0.738695	-3.149202	-2.138043
15	0.580089	-12.604513	-4.005754	0.195336	-0.357602	-0.062707
16	0.972255	-1.608460	0.600253	0.964801	0.277493	-1.224802
17	0.959086	-2.291060	0.461585	-2.664381	2.569137	1.659565
18	2.010667	-10.082104	-5.190917	-1.059575	-0.446954	-2.326596
19	0.103943	-6.117030	-2.315097	-1.526507	-2.899250	-1.641433
20	-2.576861	32.356281	14.724993	3.091315	0.883945	0.946156
21	-2.777049	37.754551	25.516749	3.571743	0.988955	1.025568
22	14.451751	-12.196783	-4.443700	-0.419243	-0.399540	0.257858
23	-3.920418	25.147060	8.151895	0.869839	1.153989	1.135090
24	-2.862470	14.411190	5.076330	0.764683	1.227369	1.615976

	7	8	9	10	11	12
7	-1.037941					
8	-4.458270	-2.995956				
9	-10.306717	-6.967546	-7.810460			
10	-0.531419	-5.004966	-6.999866	-4.242776		
11	-3.595473	-9.780010	-8.770224	-5.157166	-3.128771	
12	0.132135	-0.565473	0.319903	-0.850438	-2.290852	-4.006315
13	-2.048503	-3.511230	-3.712406	-4.421601	-0.322026	-1.998190
14	-3.034014	-1.916960	-2.822502	-1.362064	-5.643095	-4.280362
15	-2.728221	-5.079733	-2.951329	-2.313173	-0.947550	-5.434448
16	-0.185730	-0.086334	-0.569314	-1.250070	-1.013015	-3.064764
17	0.322899	-0.524414	0.200337	-0.336199	-1.567052	-3.918230
18	-0.583420	-7.867973	-3.494224	0.342576	-0.768536	-1.078915
19	-4.141995	-0.950803	-0.985658	0.196618	-0.958915	0.207990
20	4.042250	7.926229	7.620102	-1.077197	4.061529	-5.057991
21	4.246858	8.532476	8.669635	-0.422990	4.679754	-5.046764
22	-0.885005	-2.028640	-0.164688	1.452204	-1.487941	0.272454
23	-0.043410	0.221916	0.289586	-0.849418	0.873706	-0.708466
24	0.123056	-0.395348	0.458255	-0.045381	0.961182	0.322768

	13	14	15	16	17	18
13	-1.396895					
14	-2.284224	-4.348456				
15	-3.096880	-1.459721	-2.271467			
16	-1.053546	-3.633571	-5.631523	-3.430749		
17	-2.115849	-8.717298	-1.785006	-15.108760	-10.848753	
18	-7.887172	-0.561574	-0.934375	-6.635796	-2.762173	-2.874469
19	-2.556561	-4.229398	-0.343285	0.373496	-3.326552	-1.089864
20	-0.436786	0.560625	-16.847815	-2.042161	-2.373113	1.891755
21	0.526195	0.940777	-6.270721	0.197988	-0.639991	2.225516
22	-0.348850	-2.640062	-0.921450	-0.718562	-0.856943	-1.499337
23	-3.825811	-0.155355	0.822031	-7.745246	18.747664	-28.533080
24	-2.468717	-1.300113	-0.219527	-5.622562	-1.260908	-20.278009

	19	20	21	22	23	24
19	-11.132502					
20	1.226762	-26.628843				
21	1.215559	-100.291644	-34.739516			
22	-0.999644	2.817061	2.839420	-48.532613		
23	-0.292539	-5.195604	-4.963466	3.865528	-34.509775	
24	-0.834722	-3.587051	-3.574972	2.803153	-133.752410	-39.149622
